# A critical analysis of the potential for EU Common Agricultural Policy measures to support wild pollinators on farmland

**DOI:** 10.1111/1365-2664.13572

**Published:** 2020-02-16

**Authors:** Lorna J. Cole, David Kleijn, Lynn V. Dicks, Jane C. Stout, Simon G. Potts, Matthias Albrecht, Mario V. Balzan, Ignasi Bartomeus, Penelope J. Bebeli, Danilo Bevk, Jacobus C. Biesmeijer, Róbert Chlebo, Anželika Dautartė, Nikolaos Emmanouil, Chris Hartfield, John M. Holland, Andrea Holzschuh, Nieke T. J. Knoben, Anikó Kovács‐Hostyánszki, Yael Mandelik, Heleni Panou, Robert J. Paxton, Theodora Petanidou, Miguel A. A. Pinheiro de Carvalho, Maj Rundlöf, Jean‐Pierre Sarthou, Menelaos C. Stavrinides, Maria Jose Suso, Hajnalka Szentgyörgyi, Bernard E. Vaissière, Androulla Varnava, Montserrat Vilà, Romualdas Zemeckis, Jeroen Scheper

**Affiliations:** ^1^ Integrated Land Management Scotland’s Rural College Ayr UK; ^2^ Plant Ecology and Nature Conservation Group Wageningen University Wageningen The Netherlands; ^3^ University of East Anglia Norwich UK; ^4^ Department of Zoology University of Cambridge Cambridge UK; ^5^ Trinity College Dublin Dublin Ireland; ^6^ Centre for Agri‐Environmental Research School of Agriculture, Policy and Development Reading University Reading UK; ^7^ Agroecology and Environment Agroscope Zurich Switzerland; ^8^ Institute of Applied Sciences Malta College of Arts, Science and Technology (MCAST) Paola Malta; ^9^ Estación Biológica de Doñana (EBD‐CSIC) Sevilla Spain; ^10^ Department of Crop Science Agricultural University of Athens Athens Greece; ^11^ National Institute of Biology Ljubljana Slovenia; ^12^ Naturalis Biodiversity Center Leiden The Netherlands; ^13^ Institute for Environmental Sciences (CML) Universiteit Leiden Leiden The Netherlands; ^14^ Department of Poultry Science and Small Farm Animals Slovak University of Agriculture Nitra Slovakia; ^15^ Agriculture Academy of Vytautas Magnus University Akademija Lithuania; ^16^ Department of Crop Science Laboratory of Agricultural Zoology & Entomology Agricultural University of Athens Athens Greece; ^17^ National Farmers’ Union Warwickshire UK; ^18^ Game and Wildlife Conservation Trust Fordingbridge UK; ^19^ Animal Ecology and Tropical Biology, Biocenter University of Würzburg Würzburg Germany; ^20^ Lendület Ecosystem Services Research Group Institute of Ecology and Botany MTA Centre for Ecological Research Vácrátót Hungary; ^21^ Department of Entomology The Hebrew University of Jerusalem Rehovot Israel; ^22^ General Zoology Institute for Biology Martin Luther University Halle‐Wittenberg Halle (Saale Germany; ^23^ German Centre for Integrative Biodiversity Research (iDiv) Halle‐Jena‐Leipzig Leipzig Germany; ^24^ Laboratory of Biogeography & Ecology Department of Geography University of the Aegean Mytilene Greece; ^25^ ISOPlexis Gene bank University of Madeira Funchal Portugal; ^26^ Department of Biology Lund University Lund Sweden; ^27^ University of Toulouse INP INRA UMR 1248 AGIR Castanet‐Tolosan France; ^28^ Department of Agricultural Sciences, Biotechnology and Food Science Cyprus University of Technology Limassol Cyprus; ^29^ Institute for Sustainable Agriculture (IAS‐CSIC) Córdoba Spain; ^30^ Institute of Botany Faculty of Biology Jagiellonian University Kraków Poland; ^31^ INRA Avignon cedex 9 France; ^32^ Department of Agricultural Sciences, Biotechnology and Food Science Cyprus University of Technology Limassol Cyprus; ^33^ Plant Ecology and Nature Conservation Group Wageningen University Wageningen The Netherlands; ^34^ Animal Ecology Team Wageningen Environmental Research Wageningen University Wageningen The Netherlands

**Keywords:** agri‐environment schemes, bees, CAP Green Architecture, Common Agricultural Policy, Ecological Focus Areas, habitat complementarity, pollination services, pollinator conservation

## Abstract

1. Agricultural intensification and associated loss of high‐quality habitats are key drivers of insect pollinator declines. With the aim of decreasing the environmental impact of agriculture, the 2014 EU Common Agricultural Policy (CAP) defined a set of habitat and landscape features (Ecological Focus Areas: EFAs) farmers could select from as a requirement to receive basic farm payments. To inform the post‐2020 CAP, we performed a European‐scale evaluation to determine how different EFA options vary in their potential to support insect pollinators under standard and pollinator‐friendly management, as well as the extent of farmer uptake.
2. A structured Delphi elicitation process engaged 22 experts from 18 European countries to evaluate EFAs options. By considering life cycle requirements of key pollinating *taxa* (i.e. bumble bees, solitary bees and hoverflies), each option was evaluated for its potential to provide forage, bee nesting sites and hoverfly larval resources.
3. EFA options varied substantially in the resources they were perceived to provide and their effectiveness varied geographically and temporally. For example, field margins provide relatively good forage throughout the season in Southern and Eastern Europe but lacked early‐season forage in Northern and Western Europe. Under standard management, no single EFA option achieved high scores across resource categories and a scarcity of late season forage was perceived.
4. Experts identified substantial opportunities to improve habitat quality by adopting pollinator‐friendly management. Improving management alone was, however, unlikely to ensure that all pollinator resource requirements were met. Our analyses suggest that a combination of poor management, differences in the inherent pollinator habitat quality and uptake bias towards catch crops and nitrogen‐fixing crops severely limit the potential of EFAs to support pollinators in European agricultural landscapes.
5. *Policy Implications*. To conserve pollinators and help protect pollination services, our expert elicitation highlights the need to create a variety of interconnected, well‐managed habitats that complement each other in the resources they offer. To achieve this the Common Agricultural Policy post‐2020 should take a holistic view to implementation that integrates the different delivery vehicles aimed at protecting biodiversity (e.g. enhanced conditionality, eco‐schemes and agri‐environment and climate measures). To improve habitat quality we recommend an effective monitoring framework with target‐orientated indicators and to facilitate the spatial targeting of options collaboration between land managers should be incentivised.

## Graphical abstract

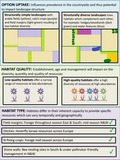

## Resumen

1. La intensificación agrícola y la consecuente pérdida de hábitats de alta calidad son desencadenantes clave del declive de los insectos polinizadores. Con el objetivo de disminuir el impacto ambiental de la agricultura, la Política Agrícola Común (PAC) de la UE de 2014 definió un conjunto de medidas para hábitats y paisajes (Áreas de Enfoque Ecológico: EFA por sus siglas en inglés) que los agricultores podían seleccionar como requisito para recibir pagos agrícolas básicos. Para informar la reforma de la PAC a partir a 2020, realizamos una evaluación a escala europea para determinar cómo las diferentes opciones de EFA varían en su potencial para asistir a los insectos polinizadores bajo un manejo estándar y amigable con los polinizadores, así como su aceptación por parte de los agricultores.
2. El proceso estructurado de elicitación Delphi para evaluar las opciones de EFA involucró a 22 expertos de 18 países europeos. Se consideraron los requisitos de los diferentes taxones de polinizadores (es decir, abejorros, abejas solitarias y sírfidos) evaluando cada opción por su potencial para proporcionar forraje, sitios de nidificación y recursos para las larvas.
3. Las opciones de EFA variaron sustancialmente en la cantidad de recursos que se percibía que proporcionan y su efectividad vario geográfica y temporalmente. Por ejemplo, los márgenes de cultivos proporcionan un forraje relativamente bueno durante toda la temporada en el sur y el este de Europa, pero carecen de forraje a principios de temporada en el norte y oeste de Europa. Bajo el manejo estándar, ninguna opción de EFA logró puntuaciones altas en todas las categorías de recursos y en general se percibió una escasez de forraje al final de la temporada.
4. Los expertos identificaron oportunidades sustanciales para mejorar la calidad del hábitat mediante la adopción de un manejo amigable con los polinizadores. Sin embargo, mejorar la gestión por sí solo es poco probable que garantice que se cumplan todos los requisitos necesarios para los polinizadores. Nuestro análisis sugiere que una combinación de manejo inadecuado, diferencias de calidad inherentes a los distintos hábitat y el sesgo de aceptación hacia cultivos de cobertura y cultivos que fijan nitrógeno limitan severamente el potencial de los EFA para apoyar a los polinizadores en los paisajes agrícolas europeos.
5. *Implicaciones políticas*. Para conservar a los polinizadores y ayudar a proteger los servicios de polinización, nuestro estudio destaca la necesidad de crear una variedad de hábitats interconectados y bien administrados que se complementen entre sí en los recursos que ofrecen. Para lograr esto, la PAC post‐2020 debe integrar los diferentes vehículos de implementación destinados a proteger la biodiversidad (por ejemplo, condicionalidad mejorada, esquemas ecológicos y medidas agroambientales y climáticas). Para mejorar la calidad del hábitat, recomendamos un marco de monitoreo efectivo con indicadores orientados a objetivos y incentivar la colaboración entre los administradores de las tierras.

## Résumé

1. L’intensification agricole et la perte associée d'habitats semi‐naturels sont les principaux moteurs du déclin des insectes pollinisateurs. Dans l'intention de réduire l'impact environnemental de l'agriculture, la politique agricole commune (PAC) de l'UE de 2014 a défini un ensemble d'habitats et d’éléments paysagers (surfaces d'intérêt écologique: SIE) dans la mise en place ou le respect desquels les agriculteurs pouvaient s'engager comme condition pour bénéficier d'aides économiques européennes (droit au paiement de base). Pour éclairer la PAC post‐2020, nous avons évalué à l'échelle européenne et à dire d'expert, d'une part les potentialités des diverses SIE à favoriser les insectes pollinisateurs, via une gestion standard et via une gestion optimisée, et d'autre part l'étendue de l'adoption de ces mesures par les agriculteurs.
2. Un processus structuré d’élaboration et d'agrégation des opinions (méthode Delphi) a fait appel à 22 experts de 18 pays européens pour évaluer les potentialités des diverses SIE. Considérant les traits bioécologiques des principaux taxons pollinisateurs (*i.e.* bourdons, abeilles solitaires et syrphes), chaque SIE a été évaluée pour son potentiel à fournir des ressources trophiques et des sites de reproduction (sites de nidification pour les bourdons et abeilles, sites de ponte et développement larvaire pour les syrphes).
3. Les SIE différaient considérablement les unes des autres sur les ressources qu'elles étaient censées offrir et leur efficacité variait géographiquement et temporellement. Par exemple, les bords de champ peuvent fournir des ressources trophiques tout au long de l'année en Europe du Sud et de l'Est mais pas en début de saison en Europe du Nord et de l'Ouest. En cas de gestion standard, aucun type de SIE n'atteint de score élevé pour aucun type de ressource, et une période de disette alimentaire survient en fin de saison.
4. Les experts ont mis en évidence de possibles et substantielles améliorations des SIE par le biais de leur gestion optimisée. Cependant, cette seule amélioration ne garantit pas la fourniture de ressources suffisantes aux pollinisateurs des paysages agricoles européens. Pour cela, des habitats spécifiques doivent être favorisés, dont la mise en place ne doit pas être entravée par un choix massif de SIE à base de cultures intermédiaires pièges à nitrates ou fixatrices d'azote.
5. *Implications politiques*. Pour préserver les pollinisateurs et le service de pollinisation des plantes entomophiles, notre étude souligne la nécessité de créer une diversité d'habitats interconnectés, gérés de façon optimale, qui se complètent mutuellement dans les ressources qu'ils offrent. Pour atteindre cet objectif, la PAC post‐2020 doit adopter une vision holistique de la mise en œuvre des différents leviers de protection de la biodiversité (e.g. éco‐conditionnalité renforcée, programmes verts ou ‘*eco‐schemes*’, mesures agro‐environnementales et climatiques). Pour réellement améliorer la qualité des habitats, nous recommandons des suivis efficaces de la biodiversité à l'aide d'indicateurs pertinents. Enfin, pour optimiser la disposition spatiale des SIE et leur connectivité, la collaboration entre les différents gestionnaires des espaces agricoles doit être encouragée.

## Samenvatting

1. De achteruitgang van bestuivende insecten in het landelijk gebied is voornamelijk het gevolg van intensivering van de landbouw en het daarmee gepaard gaande verlies van geschikt voedsel‐ en nestelhabitat. Om de impact van de landbouw op het milieu te verminderen zijn in 2014 binnen het EU Gemeenschappelijk landbouwbeleid (GLB) een aantal Ecologische Aandachtsgebieden (Ecological Focus Areas, EFAs) vastgesteld die boeren kunnen implementeren om te voldoen aan de eisen voor directe inkomenssteun. In deze studie hebben we, ten behoeve van de hervorming van het GLB voor de periode na 2020, op Europese schaal een evaluatie uitgevoerd van de potentiële waarde van verschillende EFAs voor bestuivers. De waarde van EFAs is hierbij beoordeeld onder gangbaar beheer en in het geval het beheer voor bestuivers geoptimaliseerd zou worden.
2. De evaluatie is uitgevoerd door middel van de Delphi‐methode, waarbij 22 experts uit 18 Europese landen betrokken waren. Op basis van de levenscyclusvereisten van de belangrijkste groepen bestuivende insecten (i.e. hommels, solitaire bijen en zweefvliegen) werd elk EFA habitat beoordeeld op de mate waarin het voedsel, nestgelegenheid (voor bijen), en larvaal habitat (voor zweefvliegen) kan verschaffen.
3. EFAs verschilden sterk in hun potentiële waarde voor bestuivers, en deze waarden verschilden daarnaast tussen perioden in het seizoen en tussen Europese regio's. In Zuid‐ en Oost Europa bieden gangbaar beheerde akkerranden bijvoorbeeld relatief veel voedsel gedurende het gehele seizoen, terwijl deze in Noordwest Europa relatief weinig voedsel bieden. Onder standaard beheer scoorde geen enkele EFA een hoge waarde voor alle onderzochte bestuiversgroepen en hulpbronnen, waarbij met name een gebrek aan voedsel laat in het seizoen werd vastgesteld.
4. Experts gaven aan dat meer bestuiver‐vriendelijk beheer de kwaliteit van EFAs aanzienlijk zou vergroten. Echter, zelfs onder optimaal beheer is het onwaarschijnlijk dat een enkel EFA‐type aan alle habitatvereisten van de groepen bestuivers kan voldoen. Uit onze analyses komt naar voren dat de potentie van EFAs om bestuivers in Europese agrarische landschappen te bevorderen momenteel sterk beperkt wordt door een combinatie van ongeschikt beheer en inherente verschillen in habitatkwaliteit van EFAs. Bovendien hebben de EAs die het meest door boeren worden toegepast, zoals vanggewassen en stikstofbindende gewassen, over het algemeen het minst voor bestuivers te bieden.
5. *Implicaties voor beleid*. Voor het bevorderen van bestuivers en hun bestuivingsdiensten in agrarische landschappen is het noodzakelijk om een verbonden netwerk van verschillende, goed beheerde EAs te creëren, die elkaar complementeren in het habitat dat ze voor bestuivers bieden. Om dit te bereiken is het van belang dat het GLB beleid na 2020 een holistisch aanpak van implementatie hanteert, waarbij de verschillende biodiversiteitsgerichte instrumenten (i.e. versterkte conditionaliteit, eco‐programma's, agrarisch natuurbeheer en klimaatmaatregelen) geïntegreerd worden. Daarnaast moet, om op landschapsschaal tot een ruimtelijk gebalanceerde en samenhangende implementatie van EFAs te komen, onderlinge samenwerking tussen verschillende landeigenaren worden gestimuleerd.

## Izvleček

1. Kmetijska intenzifikacija in s tem povezana izguba življenjskega prostora so glavni povzročitelji upadanja števila žuželčjih opraševalcev. Da bi zmanjšali vpliv kmetijstva na okolje, je Skupna kmetijska politika (SKP) EU 2014 določila več habitatnih in krajinskih lastnosti (površine z ekološkim pomenom ‐ PEP) izmed katerih kmetje lahko izberejo kot obveznost in zanje prejmejo plačilo. Za obveščanje o SKP po letu 2020 smo na nivoju Evrope ocenili potencial različnih PEP za podporo opraševalcem in sicer pri standardni in opraševalcem prilagojeni izvedbi in tudi glede na obseg kmetovanja.
2. V postopek pridobivanja Delphi je bilo vključenih 22 strokovnjakov iz 18 evropskih držav, ki so ocenili PEP. Z upoštevanjem potreb življenjskega kroga ključnih opraševalcev (tj. čmrljev, čebel samotark in muh trepetavk) je bila vsaka možnost ovrednotena glede potenciala zagotavljanja hrane (paše), mest za gnezdenje čebel in virov za ličinke muh trepetavk.
3. PEP so se zelo razlikovale v zagotavljanju virov, njihova učinkovitost pa se razlikuje geografsko in časovno. Na primer, robovi njiv v južni in vzhodni Evropi zagotavljajo relativno dobro pašo skozi vso sezono, medtem ko je v severni in zahodni Evropi v začetku sezone ne zagotavlja. Pri standardnem upravljanju nobena PEP ni prejela dobre ocene kot vir paše, pozno v sezoni pa so pomanjkljiv vir paše.
4. Strokovnjaki so prepoznali precejšnje možnosti za izboljšanje kakovosti habitatov s sprejetjem opraševalcem prilagojenega upravljanja. Samo izboljšanje upravljanja pa verjetno ne bi izpolnilo vseh potreb opraševalcev. Naše analize kažejo, da kombinacija slabega upravljanja, razlike v kakovosti habitatov in izbira predvsem strniščnih dosevkov in rastlin, ki fiksirajo dušik, zelo omejujejo potencial PEP za podporo opraševalcem v evropski kmetijski krajini.
5. *Posledice za politiko*. Da bi ohranili opraševalce in pomagali zaščititi storitve opraševanja, naša raziskava poudarja potrebo po ustvarjanju različnih medsebojno povezanih in dobro upravljanih habitatov, ki se med seboj dopolnjujejo v virih, ki jih ponujajo. Da bi to dosegli, bi morala SKP po letu 2020 zavzeti celosten pogled na izvedbo, ki združuje različne ukrepe za zaščito biotske raznovrstnosti (npr. večja povezanost kmetijsko‐okoljskih in podnebnih ukrepov). Za izboljšanje kakovosti habitatov priporočamo učinkovit monitoring s ciljno usmerjenimi kazalci, za pomoč pri varovanju ciljnih habitatov, pa bi bilo treba vzpodbuditi sodelovanje med upravljavci zemljišč.

## Abstrakt

1. Die Intensivierung der Landwirtschaft und der damit verbundene Verlust an hochwertigen Lebensräumen sind die Hauptursachen für den Rückgang der Insektenbestäuber. Mit dem Ziel, die Umweltauswirkungen der Landwirtschaft zu verringern, wurde in der Gemeinsamen Agrarpolitik (GAP) der EU 2014 eine Reihe von Lebensraum‐ und Landschaftsmerkmalen (Ökologische Schwerpunktbereiche: ‚Ecological Focus Areas‘ oder EFA) definiert, aus denen die Landwirte als Voraussetzung für den Erhalt von Basisprämien für landwirtschaftliche Betriebe auswählen können. Um die zukünftige GAP ab 2020 zu informieren, haben wir eine europaweite Bewertung durchgeführt, um zu ermitteln, wie sich die verschiedenen EFA‐Optionen in ihrem Potenzial zur Unterstützung von Insektenbestäubern unter standardmäßigem und bestäuberfreundlichem Management unterscheiden und in welchem Umfang die Landwirte sie annehmen.
2. In einem strukturierten Delphi‐Erfassungsprozess wurden 22 Experten aus 18 europäischen Ländern mit der Bewertung von EFA‐Optionen beauftragt. Unter Berücksichtigung der Lebenszyklusanforderungen der wichtigsten Bestäubertaxa (d.h. Hummeln, Solitärbienen und Schwebfliegen) wurde jede Option auf ihr Potenzial zur Bereitstellung von Nahrung, Bienennistplätzen und Ressourcen für Schwebfliegenlarven bewertet.
3. Die EFA‐Optionen unterschieden sich erheblich in den Ressourcen, die sie nach allgemeiner Auffassung bereitstellen, und ihre Wirksamkeit variierte geografisch und zeitlich. Beispielsweise bieten die Feldränder in Süd‐ und Osteuropa während der gesamten Saison relativ gutes Futter, während es in Nord‐ und Westeuropa zu Beginn der Saison an Futter fehlt. Unter der Standardbewirtschaftung erzielte keine einzige EFA‐Option hohe Werte über alle Ressourcenkategorien hinweg, und eine Knappheit an Futter in der Spätsaison konnte wahrgenommen werden.
4. Die Experten erkannten erhebliche Möglichkeiten zur Verbesserung der Lebensraumqualität durch ein bestäuberfreundliches Management. Eine Verbesserung des Managements allein dürfte jedoch nicht gewährleisten, dass alle Anforderungen an die Bestäuberressourcen erfüllt werden. Unsere Analysen deuten darauf hin, dass eine Kombination aus schlechtem Management, Unterschieden in der inhärenten Qualität der Bestäuberhabitate und einer Verzerrung der Aufnahme von Zwischenfrüchten und stickstofffixierenden Kulturen das Potential der EFAs zur Unterstützung der Bestäuber in den europäischen Agrarlandschaften stark einschränkt.
5. *Auswirkungen auf die Politik*. Um die Bestäuber zu erhalten und zum Schutz der Bestäubungsdienste beizutragen, unterstreicht unsere Studie die Notwendigkeit, eine Vielzahl miteinander verbundener, gut verwalteter Lebensräume zu schaffen, die sich in ihren Ressourcen gegenseitig ergänzen. Um dies zu erreichen, sollte die GAP nach 2020 eine ganzheitliche Sichtweise der Umsetzung einnehmen, die die verschiedenen Instrumente zum Schutz der biologischen Vielfalt integriert (z.B. verbesserte Auflagen, Öko‐Regelungen und Agrar‐Umwelt‐ und Klima‐Maßnahmen). Zur Verbesserung der Habitatqualität empfehlen wir einen wirksamen Überwachungsrahmen mit zielorientierten Indikatoren, und um die räumliche Ausrichtung der Optionen zu erleichtern, und um die Zusammenarbeit zwischen den Landmanagern zu fördert.

## Περίληψη

1. Η εντατικοποίηση της γεωργίας, και κατά συνέπεια η απώλεια ενδιαιτημάτων υψηλής ποιότητας, αποτελούν τους βασικούς λόγους μείωσης των εντόμων επικονιαστών. Με σκοπό την ελάφρυνση των περιβαλλοντικών επιπτώσεων στη γεωργία, η Κοινή Αγροτική Πολιτική (ΚΑΠ) του 2014 της Ευρωπαϊκής Ένωσης καθόρισε μια ομάδα οικοσυστημικών και τοπιακών χαρακτηριστικών (Περιοχές Οικολογικής Εστίασης: ΠΟΕ) που απαιτείται από τους γεωργούς να επιλέξουν ώστε να τύχουν βασικής χρηματοδότησης για τα αγροκτήματά τους. Στοχεύοντας στην πληροφόρηση της μετά το 2020 ΚΑΠ, αξιολογήσαμε σε πανευρωπαϊκό επίπεδο τις διάφορες εκδοχές των ΠΟΕ, με σκοπό τον καθορισμό της δυνατότητάς τους να υποστηρίζουν τα έντομα επικονιαστές υπό συνθήκες κανονικής (συνήθους) διαχείρισης και διαχείρισης φιλικής προς τους επικονιαστές.
2. Εφαρμόσθηκε μια δομημένη διαδικασία της Τεχνικής των Δελφών, με συμμετοχή 22 ειδικών από 18 Ευρωπαϊκές χώρες, οι οποίοι αξιολόγησαν τις εκδοχές των ΠΟΕ. Λαμβάνοντας υπόψιν τις απαιτήσεις των σημαντικών ομάδων επικονιαστών εφ’ όλου του κύκλου ζωής τους (βομβίνοι, μοναχικές μέλισσες και συρφίδες), κάθε εκδοχή αξιολογήθηκε για την δυνατότητά της να προσφέρει νομή, περιοχές φωλιάσματος για τις μέλισσες και απαιτούμενους πόρους για τις συρφίδες.
3. Οι διάφορες εκδοχές των ΠΟΕ διέφεραν σημαντικά ως προς τους πόρους που αναμένονταν να προσφέρουν, με αποτελεσματικότητα που ποίκιλλε γεωγραφικά και χρονικά. Για παράδειγμα, τα όρια των χωραφιών προσφέρουν σχετικά καλή νομή καθ’ όλη τη διάρκεια της παραγωγικής περιόδου στη Νότιο και Ανατολική Ευρώπη, κάτι που δεν συμβαίνει νωρίς την άνοιξη στην Βόρειο και Δυτική Ευρώπη. Υπό συνήθεις συνθήκες διαχείρισης, καμία εκδοχή ΠΟΕ δεν έλαβε υψηλή βαθμολογία για τις διάφορες κατηγορίες εξεταζόμενων πόρων, ενώ παρατηρήθηκε μειωμένη ανθονομή προς το τέλος της παραγωγικής περιόδου.
4. Οι ειδικοί αναγνώρισαν την ύπαρξη σημαντικών ευκαιριών βελτίωσης της ποιότητας των ενδιαιτημάτων με την υιοθέτηση διαχείρισης φιλικής προς τους επικονιαστές. Η απλή, πάντως, βελτίωση διαχείρισης θεωρήθηκε απίθανο να διασφαλίσει τις απαιτήσεις πόρων για όλους τους επικονιαστές. Οι αναλύσεις μας δείχνουν ότι ο συνδυασμός κακής διαχείρισης, οι διαφορές ως προς την ποιότητα ενδιαιτήματος για τους υπάρχοντες επικονιαστές και η επιλογή ανάπτυξης παρεμβαλλόμενων και αζωτοδεσμευτικών καλλιεργειών περιορίζουν σοβαρά την δυνατότητα των ΠΟΕ να υποστηρίξουν επικονιαστές στα Ευρωπαϊκά γεωργικά τοπία.
5. *Συμπεράσματα για άσκηση πολιτικών*. Για την διατήρηση των επικονιαστών και την προστασία των υπηρεσιών επικονίασης, η έρευνά μας υπογραμμίζει την ανάγκη δημιουργίας μιας ποικιλίας αλληλοσυνδεόμενων ενδιαιτημάτων υπό καθεστώς καλής διαχείρισης, τα οποία είναι συμπληρωματικά ως προς τους πόρους που προσφέρουν. Προς αυτή την κατεύθυνση, η μετά το 2020 ΚΑΠ θα πρέπει να υιοθετήσει μια ολιστική οπτική υλοποίησης, η οποία θα ενοποιεί τα διάφορα μέσα για την προστασία της βιοποικιλότητας (π.χ. επαυξημένη υποθετικότητα, οικολογικά σχέδια, Αγρο‐Περιβαλλοντικά και Κλιματικά Μέτρα). Για την βελτίωση της ποιότητας των ενδιαιτημάτων συνιστούμε ένα αποτελεσματικό σχήμα παρακολούθησης (monitoring) με στοχοθετημένους δείκτες. Τέλος, θεωρούμε ότι η χωρική εφαρμογή των διάφορων εκδοχών ΠΟΕ θα διευκολυνθεί με την υιοθέτηση κινήτρων που θα προάγουν την συνεργασία μεταξύ των διαχειριστών γης.

## Santrauka

1. Žemės ūkio suintensyvėjimas ir su tuo susijęs vertingų buveinių praradimas yra pagrindiniai vabzdžių apdulkintojų nykimo veiksniai. Siekiant sumažinti žemės ūkio poveikį aplinkai, 2014 m. ES bendrojoje žemės ūkio politikoje (BŽŪP) buvo apibrėžtos buveinių ir kraštovaizdžio elementų nustatymo gairės (ekologiniu atžvilgiu svarbios vietovės (EASV)), kurias ūkininkai galėjo pasirinkti kaip reikalavimą gauti pagrindines ūkio išmokas. Siekdami informuoti BŽŪP po 2020 m., atlikome vertinimą Europos mastu, siekdami išsiaiškinti, skiriasi EASV variantai, atsižvelgiant į jų reikšmę vabzdžiams apdulkintojams, taikant įprastą ir apdulkintojams palankų valdymą, bei ūkininkų galimybes įsitraukti į šias veiklas.
2. Struktūrizuotame „Delphi“ elicitacijos procese, vertindami EASV galimybes, dalyvavo 22 ekspertai iš 18 Europos šalių. Atsižvelgiant į pagrindinių apdulkintojų taksonus (t.y. kamanių, bičių vienišių ir žiedmusių) gyvenimo ciklo reikalavimus, kiekviena EASV buvo įvertinta atsižvelgiant į jos galimybes aprūpinti pašarą, suteikti vietų bičių lizdavietėms ir žiedmusių lervų išteklius.
3. EASV galimybės labai skyrėsi atsižvelgiant į išteklius, kuriuos, jų manymu, buvo numatyta suteikti, o jų veiksmingumas skyrėsi geografiškai ir laikinai. Pvz., lauko pakraščiai buvo įvertinti kaip gana geros ganyklos visą sezoną Pietų ir Rytų Europoje, tačiau trūko ankstyvųjų ganyklų Šiaurės ir Vakarų Europoje. Taikant įprastinę praktiką, nė vienas EASV variantas nepasiekė aukštų išteklių kategorijų balų ir akivaizdžiai pasireiškė vėlyvojo sezono ganyklų trūkumas.
4. Ekspertai nustatė dideles galimybes pagerinti buveinių kokybę, taikant apdulkintojams palankų tvarkymą. Tačiau ėmusis tik palankaus tvarkymo, nebus užtikrinti visi apdulkintojų poreikiai. Mūsų analizė rodo, kad netinkamas tvarkymas, būdingų apdulkintojų buveinių kokybės skirtumai, tarpinių pasėlių ir azotą fiksuojančių pasėlių įsivyravimas smarkiai riboja EASV galimybes pagerinti apdulkintojų būklę Europos žemės ūkio kraštovaizdyje.
5. *Politikos padariniai*. Norėdami išsaugoti apdulkintojus ir padėti apsaugoti apdulkinimo paslaugas, mūsų tyrimas pabrėžia poreikį sukurti įvairias tarpusavyje sujungtas, gerai tvarkomas buveines, papildančias viena kitą jų siūlomais ištekliais. Norint tai pasiekti, BŽŪP po 2020 m. turėtų būti holistinis požiūris į įgyvendinimą, integruojantį skirtingas priemones, kuriomis siekiama apsaugoti biologinę įvairovę (pvz., geresnės sąlygos, ekologinės schemos ir agrarinės aplinkosaugos bei klimato priemonės). Norėdami pagerinti buveinių kokybę, mes rekomenduojame veiksmingą stebėsenos sistemą su tiksliniais rodikliais ir siekiant palengvinti erdvinį tikslinį pasirinkimą, turėtų būti skatinamas žemės valdytojų bendradarbiavimas.

## Abstrakt

1. Intensyfikacja rolnictwa i związana z nim utrata wysokiej jakości siedlisk są kluczowymi czynnikami zaniku owadów zapylających. W celu zmniejszenia negatywnego wpływu rolnictwa na środowisko, wspólna polityka rolna UE w 2014 r. (WPR) zdefiniowała zestaw cech siedlisk i krajobrazów (obszary proekologiczne, tzw. obszary EFA), z których rolnicy mogliby wybrać elementy do utrzymania jako podstawowy warunek otrzymania płatności dla gospodarstw rolnych. W celu dostarczenia informacji dla WPR po 2020 r., przeprowadziliśmy ocenę na skalę europejską, aby określić w jaki sposób różne opcje EFA różnią się pod względem potencjału wspierania owadów zapylających w ramach standardowego i przyjaznego dla zapylaczy zarządzania, a także stopnia wykorzystania przez rolników.
2. W ustrukturyzowanym procesie pozyskiwania Delphi uczestniczyło 22 ekspertów z 18 krajów europejskich w celu oceny wariantów obszarów proekologicznych. Biorąc pod uwagę wymagania dotyczące cyklu życia kluczowych taksonów zapylających (tj. trzmieli, samotnych pszczół i bzygów), każda opcja została oceniona pod kątem możliwości zapewnienia pożytku, miejsc gniazdowania pszczół i zasobów dla larw bzygów.
3. Opcje EFA różniły się znacznie pod względem zasobów, które ‐ jak sądzono ‐ zapewniały, a ich skuteczność była zróżnicowana geograficznie i czasowo. Na przykład obrzeża pól uprawnych zapewniają stosunkowo dobry pożytek przez cały sezon w Europie Południowej i Wschodniej, ale brakowało tam wczesnego pożytku w Europie Północnej i Zachodniej. W ramach standardowego zarządzania żadna pojedyncza opcja EFA nie osiągnęła wysokich wyników we wszystkich kategoriach zasobów i zauważono niedobór pożytku pod koniec sezonu.
4. Eksperci zidentyfikowali znaczne możliwości poprawy jakości siedlisk poprzez przyjęcie przyjaznego dla zapylaczy zarządzania. Samo usprawnienie zarządzania nie zapewniło jednak spełnienia wszystkich wymagań dotyczących zasobów zapylaczy. Nasze analizy sugerują, że połączenie złego zarządzania, różnic w jakości siedlisk dla zapylaczy i tendencyjności absorpcji w kierunku upraw międzyplonów i upraw wiążących azot poważnie ogranicza potencjał obszarów proekologicznych do wspierania zapylaczy w europejskich krajobrazach rolniczych.
5. *Implikacje polityczne*. Aby chronić zapylaczy i pomóc chronić usługi zapylania, nasze badanie podkreśla potrzebę stworzenia różnorodnych, połączonych, dobrze zarządzanych siedlisk, które uzupełniają się nawzajem w oferowanych zasobach. Aby to osiągnąć, WPR po 2020 r. powinna uwzględniać holistycznie podejście do wdrażania, które integruje różne narzędzia dostarczania mające na celu ochronę różnorodności biologicznej (np. zwiększoną warunkowość, ekoschematy oraz działania rolnośrodowiskowe i klimatyczne). Aby poprawić jakość siedlisk, zalecamy skuteczne monitorowania ze wskaźnikami zorientowanymi na powyższe cele oraz w celu ułatwienia ukierunkowania działań w przestrzeni należy zachęcać do współpracy między zarządcami gruntów.

## Sumário

1. A intensificação agrícola e a perda associada de habitats de elevada qualidade são os principais factores que impulsionam o declínio dos insetos polinizadores. A fim de mitigar o impacto ambiental da agricultura, a Política Agrícola Comum (PAC) da UE, de 2014, definiu um conjunto de atributos ou estruturas do habitat e da paisagem, designadas de Áreas Foco Ecológico (AFEs) que devem ser mantidas pelos agricultores como requisito para obter as ajudas económicas previstas nas medidas agroambientais. No presente trabalho realizamos uma avaliação à escala europeia das diferentes opções destas estruturas, a fim de munir a PAC pós‐2020, com informação sobre a importância das AFEs. Estas variam muito quanto ao seu potencial no apoio às populações de polinizadores, de acordo com a extensão da sua aceitação pelos agricultores e das práticas adoptadas por estes na sua gestão, que podem consistir em práticas padrão ou práticas mais amigáveis para os polinizadores.
2. Um processo estruturado, com base na técnica de elicitação de Delphi foi desenvolvido, envolvendo 22 especialistas de 18 países europeus, com o objectivo de avaliar as opções de AFEs previstas na PAC. Esta avaliação levou em consideração os requisitos do ciclo de vida dos taxa dos principais polinizadores, ou seja, as abelhas, as abelhas solitárias e os sirfídeos ou moscas‐das‐flores. Cada AFE foi avaliada quanto ao seu potencial para fornecer alimento, locais de nidificação, e recursos para as larvas dos sirfídeos.
3. A percepção quanto à eficácia das AFEs como fonte de recursos (alimento) para os polinizadores variou substancialmente, do ponto de vista quer geográfico, quer temporal (época do ano). Por exemplo, a AFE, faixas verdes nas margens do campo são consideradas uma boa fonte de alimento, no sul e leste da Europa, durante todo ano, mas ineficazes, no norte e oeste da Europa, no início do ano. Nenhuma EFA alcançou pontuações elevadas na categoria de recursos (fonte de alimento), quando submetida ao maneio padrão, sendo consideradas ineficientes, na segunda metade do ano.
4. Os especialistas envolvidos identificaram oportunidades de melhoria substancial na qualidade do habitat, através da adopção de práticas de maneio das EFAs mais “amigáveis” para com os polinizadores. No entanto, a melhoria das práticas de maneio das EFAs por si só, dificilmente garantirá todos os requisitos necessários para a manutenção das populações de polinizadores. A nossa avaliação sugere que a combinação de práticas de má gestão (maneio), diferenças inerentes à qualidade do habitat dos polinizadores e o aumento do bias que resulta da utilização de espécies de crescimento rápido ou fixadoras de azoto limitam severamente o papel e potencial destas estruturas na manutenção das populações de polinizadores nas paisagens agrícolas europeias.
5. *Implicações políticas*. A conservação dos polinizadores ajuda a proteger os serviços de polinização providenciados por estes. O nosso estudo destaca a necessidade de criar uma variedade de habitats interconectados e geridos de forma que se complementem na oferta de recursos (alimento, locais de nidificação e recursos para as larvas) aos polinizadores. Para atingir este objectivo, a PAC pós‐2020 deve adoptar uma visão holística na implementação das EFAs, que integre os diferentes programas destinados a protecção da biodiversidade (por exemplo, maior condicionalidade, esquemas ecológicos, e medidas agroambientais e de adaptação climática). Para melhorar a qualidade do habitat, recomendamos uma estrutura de monitorização eficaz suportada por indicadores quantitativos e qualitativos orientados para metas, que permitam facilitar a tomada de decisões direcionadas especificamente para as EFAs, e que a colaboração entre os gestores da terra (agricultores) seja incentivada.

## Összefoglaló

1. A mezőgazdasági intenzifikáció és ennek következtében az értékes, természetközeli élőhelyek eltűnése a beporzó rovarok csökkenéséhez nagyban hozzájárul. A mezőgazdaság környezeti hatásainak mérséklése érdekében az Európai Unió 2014‐es Közös Agrárpolitikai reformja (KAP) meghatározott egy sor olyan élőhelyi vagy táji elemet (ökológiai fókusz területek, angol nevén *Ecological Focus Areas* ‐ EFA), melyekből létesítéséből/megőrzéséből a mezőgazdasági gazdák választhatnak az alaptámogatásban való részesülés feltételeként. Most a 2020 utáni KAP kidolgozásához egy európai léptékű értékelést készítettünk a különböző EFA lehetőségek beporzók szempontjából tekintett hasznosságáról, az alapértelmezett és beporzók számára kedvezőbb kezelési módok esetén.
2. Tizennyolc európai országból 22 szakértő vett részt az EFA értékelésében Delphi módszer segítségével. A kulcsfontosságú beporzó csoportok (poszméhek, magányos vadméhek, zengőlegyek) életfeltételeinek figyelembe vételével minden EFA lehetőség esetén értékeltük a táplálék‐ (virág‐) források mennyiségét, méhek esetében a fészkelési alkalmasságot, zengőlegyeknél külön a lárvák számára elérhető táplálékot is.
3. Az EFA lehetőségek jelentősen eltértek a beporzó rovarok számára fontos források biztosítása szempontjából, és becsült hatékonyságuk térben és időben is változott. Például a táblaszegélyek viszonylag jó táplálkozási lehetőségeket biztosítanak tavasztól őszig Dél‐ és Kelet‐Európában, de virágokban szegényebbek a tavaszi időszakban Észak és Nyugat‐ Európában. A standard művelés mellett egyetlen EFA sem kapott magas értéket egyik forrástípus esetén sem és a nyárvégi‐őszi időszakban forráshiányosnak bizonyultak.
4. A szakértők lényegi lehetőségeket határoztak meg az élőhelyek minőségének javítása érdekében egy beporzó‐barát művelés útján. A művelés ezirányú fejlesztése önmagában azonban valószínűleg még nem biztosítja az összes beporzó számára fontos források meglétét. Az elemzéseink alapján a nem megfelelő művelés, a beporzók számára szükséges különböző élőhelyi lehetőségek és az ökológiai jelentőségű másodvetés vagy nitrogénmegkötő növényekkel bevetett területek túlzott használata más EFA lehetőségekhez képest lényegesen csökkenti az EFA‐ban rejlő potenciált a beporzók megőrzésére az európai mezőgazdasági tájban.
5. *Következtetések, szakpolitikai hatások*. A kutatásunk rámutat a különféle, térben változatos, megfelelően művelt területek, élőhelyek létesítésének fontosságára a beporzók megőrzése és a beporzás segítése érdekében, melyek egymást kiegészítve nyújtanak különböző forrásokat. Ehhez a 2020 utáni KAP részéről is holisztikus megközelítésre van szükség, amely egybegyúrja a különböző, élőlények sokféleségének megőrzését célzó eszközöket (pl. megerősített feltételrendszer, ökológiai gazdálkodás, agrár‐környezetvédelmi és klímavédelmi programok eszközrendszere). Az élőhelyek minőségének javításához egy hatékony monitoring keretrendszert javaslunk, célorientált indikátorokkal és a megfelelő eszközök megfelelő helyen való alkalmazásával, a földhasználók közti együttműködés ösztönzésével.

## Abstrakt

1. Medzi hlavné faktory poklesu opeľujúceho hmyzu patrí intenzifikácia poľnohospodárstva a s ňou spojený úbytok vysoko kvalitných biotopov. V snahe znížiť vplyv poľnohospodárstva na životné prostredie bol v rámci Spoločnej poľnohospodárskej politiky EÚ (SPP) v roku 2014 zadefinovaný súbor biotopov a krajinných prvkov (oblastí ekologického záujmu: EFA), z ktorých si poľnohospodári môžu vyberať pre splnenie požiadaviek získania základných poľnohospodárskych platieb. Za účelom poskytnutia informácií pre SPP po roku 2020 sme vyhodnotili rozdiely jednotlivých možností EFA na európskej úrovni vzhľadom na ich potenciál podporovať výskyt opeľujúceho hmyzu pri štandardnom a ku opeľovačom šetrnom hospodárení, ako aj rozsah ich využívania poľnohospodármi.
2. Posudzovania jednotlivých možností EFA sa zúčastnilo 22 odborníkov z 18 európskych krajín s využitím Delfskej metódy štandardizovaných dotazníkov. Zohľadniac špecifiká životného cyklu hlavných skupín opeľujúceho hmyzu (čmeľov, samotárskych včiel a pestríc) bol pri každej z možností vyhodnotený jej potenciál poskytovať opeľovačom potravinové zdroje, hniezdne príležitosti, resp. zdroje pre vývoj lariev pestríc.
3. Možnosti poskytovaných zdrojov, ktoré boli pri jednotlivých EFA očakávané, sa značne líšili a aj ich využiteľnosť bola geograficky a časovo rozdielna. Multifunkčné okraje polí napríklad celoročne poskytujú dostatok potravy v južných a východných častiach Európy, v severnej a západnej Európe však neposkytujú skorú jarnú znášku. Pri štandardnom obhospodarovaní nedosiahla žiadna z možností EFA vysoké skóre vo všetkých z hodnotených kategórií a bol zaznamenaný nedostatok potravných zdrojov na konci sezóny.
4. Odborníci identifikovali značné príležitosti na zlepšenie kvality biotopu prijatím zásad obhospodarovania priateľského voči opeľovačom. Samotné zlepšenie obhospodarovania však samo o sebe nezabezpečí splnenie všetkých nárokov opeľovačov. Naše analýzy naznačujú, že kombinácia zlého hospodárenia, rozdielov v kvalite prirodzeného biotopu opeľovačov a preferovanie plodín viažucich dusík výrazne obmedzujú potenciál EFA na podporu výskytu opeľovačov v európskej poľnohospodárskej krajine.
5. *Odporúčania pre tvorcov politík*. V záujme ochrany opeľovačov a opeľovacieho servisu naša štúdia zdôrazňuje potrebu vytvoriť celý rad vzájomne prepojených, dobre spravovaných biotopov, ktoré sa navzájom dopĺňajú v sortimente poskytovaných zdrojov. Na dosiahnutie tohto cieľa by mala SPP po roku 2020 zabezpečiť celistvejší pohľad na implementáciu, integrujúc rôzne nástroje ovplyvňujúce ochranu biodiverzity (napr. zlepšovanie krížového plnenia, systémov ekologického poľnohospodárstva, agroenvironmentálnych a klimatických opatrení). Na zlepšenie kvality biotopov odporúčame zaviesť efektívny monitorovací rámec s cieľovo orientovanými ukazovateľmi a na zlepšenie priestorového zacielenia jednotlivých opatrení zintenzívnenie spolupráce s poľnohospodármi.

## 1 INTRODUCTION

Since the 1950s, agricultural biodiversity has undergone significant declines globally (Benton, Vickery, & Wilson, 2003). The intensification of agricultural practices and associated loss of high‐quality habitats, both within the crop and adjacent (semi)‐natural land, are amongst the primary drivers of biodiversity loss (Benton et al., 2003; IPBES, 2019). Farmland biodiversity underpins a range of ecosystem services vital to both natural and farmed ecosystems, including nutrient cycling, natural pest regulation and pollination, with losses indirectly constraining agricultural productivity (Deguines et al., 2014) and impacting on (semi)‐natural habitats (Ollerton, Winfree, & Tarrant, 2011; Potts et al., 2016).

To mitigate adverse environmental impacts of intensive agriculture, the European Union's Common Agricultural Policy (CAP) introduced agri‐environment schemes in 1992 to financially support environmentally friendly farming practices (EEC Regulation No 2078/92). Unfortunately, the success and cost‐effectiveness of such schemes at halting biodiversity declines remains debatable (Batáry, Dicks, Kleijn, & Sutherland, 2015; Pe’er, Lakner, et al., 2017). Consequently, to improve environmental sustainability, the 2014 CAP reform linked basic farm payments (i.e. ‘direct payments’ and ‘market‐related expenditures’) to compulsory greening measures (EU Regulation No 1307/2013). Three greening measures were introduced: maintenance of permanent pastures, crop diversification and Ecological Focus Areas (EFAs; European Commission, 2017). EFAs specifically aimed to provide ecologically beneficial areas within arable cropping systems to *safeguard and improve biodiversity on farms* (European Commission, 2017).

Proposals for the post‐2020 CAP (budget period: 2021–2027) outline plans to abandon EFAs in their current format (European Commission, 2019). Instead, it is proposed that Member States set a *minimum share of agricultural area devoted to non‐productive features or areas* as part of obligatory standards for good agricultural and environmental condition of the land, with the threshold area and available landscape/habitat options being set by Member States. In principle, this proposition is similar to current EFA requirements; however, with implementation being determined by individual Member States, recommendations on the minimum area, management and relative environmental and conservation value of different options are lacking.

Pollinators provide key services to insect‐pollinated crops and wild plants across Europe, yet they are vulnerable to agricultural intensification and habitat loss (Potts et al., 2016). Indeed, a pan‐European study of pollination potential indicated a deficit for large parts of northern Europe (Zulian, Maes, & Paracchini, 2013). Pollinators may forage in crop habitats during the short period when crops flower, but the rest of the year they rely on surrounding semi‐natural habitats for vital resources: food, shelter, nesting, breeding and dormancy/overwintering sites (Baude et al., 2016; Kovács‐Hostyánszki et al., 2017). Local and landscape structures influence the abundance and diversity of insects visiting pollinator‐dependent crops, directly impacting yield (Blaauw & Isaacs, 2014; Garibaldi et al., 2016). With animal pollinators benefitting production in approximately 75% of major crops world‐wide (Klein et al., 2007), maintaining healthy pollinator communities is critical to food security. Furthermore, with an estimated >87.5% of flowering plant species benefitting from animal pollination world‐wide, pollinator conservation is fundamental to the preservation of wider biodiversity (Ollerton et al., 2011).

Through providing habitats and enhancing landscape heterogeneity, EFAs have the potential to increase the abundance, diversity and spatio‐temporal continuity of vital resources for pollinators in agricultural landscapes. However, the success of EFAs at meeting biodiversity goals has been fiercely challenged, largely as a result of high proportion of farms being exempt and uptake bias towards more production‐orientated EFAs (European Court of Auditors, 2017; Hart et al., 2017; Pe’er, Zinngrebe, et al., 2017). EFA options vary greatly in their effects, and, because their environmental efficacy is largely dependent on the way in which they are implemented and managed, these effects can differ geographically (Alliance Environment & Thünen Institute, 2017). The post‐2020 CAP reform provides an opportunity to improve implementation of non‐productive features/areas and to outline management recommendations targeted to farm or regional requirements (e.g. diffuse pollution mitigation, pollinator conservation).

Here we provide a critical evaluation of how different EFA options can support pollinators by considering their inherent potential to provide key resources, their management and their uptake. We focus on important pollinators, specifically bees (Hymenoptera: Apiformes) and hoverflies (Diptera: Syrphidae). For each EFA option, we identify standard and ‘pollinator‐friendly’ (i.e. enhanced actions specifically designed to increase the availability of resources for pollinators) management practices. With comprehensive empirical data on the relative value of EFA options to provide pollinator resources (i.e. forage, bee nesting and hoverfly larval resources) lacking, we use a Delphi expert elicitation process to evaluate EFAs (Mukherjee et al., 2015). Our European‐scale evaluation aims to answer the following questions to inform the CAP post‐2020 on key measures to promote pollinator conservation on farmland:

1. How do EFA options differ in their potential to provide pollinator resources and how does this vary temporally (through the year) and geographically (across Europe)?
2. To what extent does improving the management of EFAs enhance their quality in terms of the range and quantity of resources offered?
3. Do different EFAs complement each other in the type and spatio‐temporal distribution of resources they offer, and could this complementarity be exploited by encouraging farmers to take up particular combinations of options?

**Figure 1:**
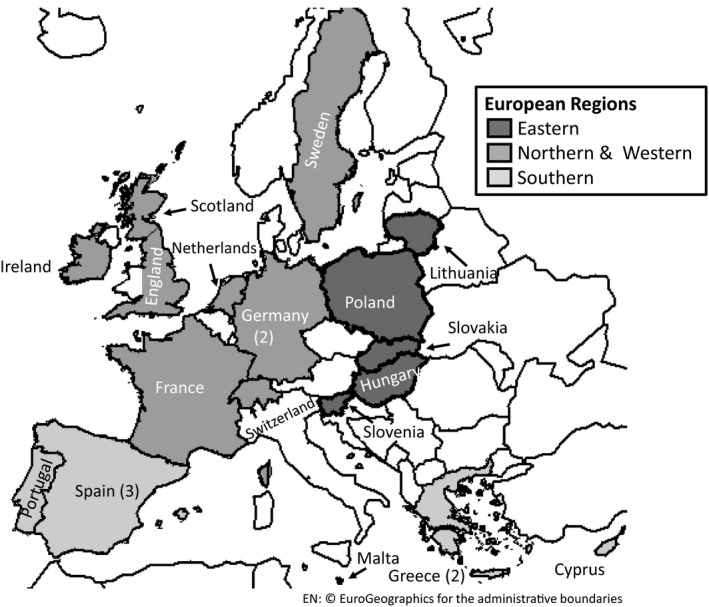
Overview of our three European geographical regions and countries represented in each region. Geographical regions were based on Köppen‐Geiger Climate Regions (Kottek et al., 2006). For countries where more than one expert scored the number of scorers is represented in brackets

Through answering these key questions, and subsequent analyses, we derive implications for EFAs, for Agri‐Environment Schemes and for the ‘Green Architecture’ of the CAP.

## 2 MATERIALS AND METHODS

### 2.1 Evaluation process

EFA options were evaluated following the Delphi technique (see Figure S1) which seeks consensus of expert opinion via anonymous, iterative rounds of evaluations and reduces bias that can accompany expert judgement (e.g. subjectivity, overconfidence, social pressure, group‐thinking and dominance: Mukherjee et al., 2015). First, a workshop was held to bring pollinator experts from across Europe together. Participants discussed ‘standard’ (i.e. typical of EFAs across regions) and ‘pollinator‐friendly’ (i.e. enhanced management designed to increase pollinator resources) management practices, identified nine important resources for key pollinator *taxa* (i.e. hoverflies, bumble bees and solitary bees: Table 1) and provided feedback on the proposed scoring document (an evaluation spreadsheet). A scientific literature review was then undertaken to provide detailed descriptions of EFA options (Table S1), summarize what is known about each option's potential to provide pollinator resources and refine the definitions of pollinator‐friendly and standard management (Table S2 outlines standard and pollinator‐friendly management including, for each EFA, comprehensive recommendations for pollinator‐friendly management).

**Table 1:** Description of insect pollinator resources included in the evaluation process

Pollinator resource	Resource description
Floral	
Early season	Flowers that provide nectar and/or pollen resources early in the year (i.e. European spring)
Mid‐season	Flowers that provide nectar and/or pollen resources towards the middle of the year (i.e. early summer/mid‐summer depending on region)
Late season	Flowers that provide nectar and/or pollen resources late in the year (i.e. late summer/autumn depending on region)
Open flowers easily accessible	Flowers that are easily accessible to most pollinator species including those with short mouthparts (e.g. *Crataegus monogyna* and *Valeriana officinalis*)
Tubular flowers accessible by long‐tongued species	Flowers that are complex in structure with deep corollae where access is restricted to long‐tongued pollinators (e.g. *Symphytum officinale* and *Vicia faba*)
Bee nesting	
Solitary bees	Suitable nesting sites for solitary bees, such as bare ground, cavities in trees, plants or man‐made structures
Bumble bees	Suitable nesting sites for bumble bees, such as tussocky grasses, old mammal burrows
Hoverfly larvae	
Insectivorous larvae	Suitable prey items (particularly aphids) for insectivorous hoverfly larvae such as *Syrphus* spp. and *Episyrphus* spp.
Saprophytic larvae	Damp, decaying organic matter that provides a food source for hoverflies with saprophytic larvae such as *Helophilus* spp. and *Eristalis s*pp.

The formal Delphi process engaged 22 experts from 18 European countries which were divided into three broad Köppen‐Geiger Climate Regions specifically: Northern and Western (N&W), Southern (S) and Eastern (E) Europe (Figure 1; Kottek, Grieser, Beck, Rudolf, & Rubel, 2006). To provide sufficient replication each Köppen‐Geiger region was represented by a minimum of five countries. To ensure anonymity of responses, evaluation spreadsheets were distributed and collated via email by a central administrator not involved in the scoring exercise. Experts were requested to evaluate all EFA habitats physically present in their country (i.e. irrespective of whether the habitat was a permitted EFA option in that country). As Switzerland is not in the EU, our Swiss evaluator was only requested to score agri‐environment habitats comparable to European EFAs.

**Figure 2:**
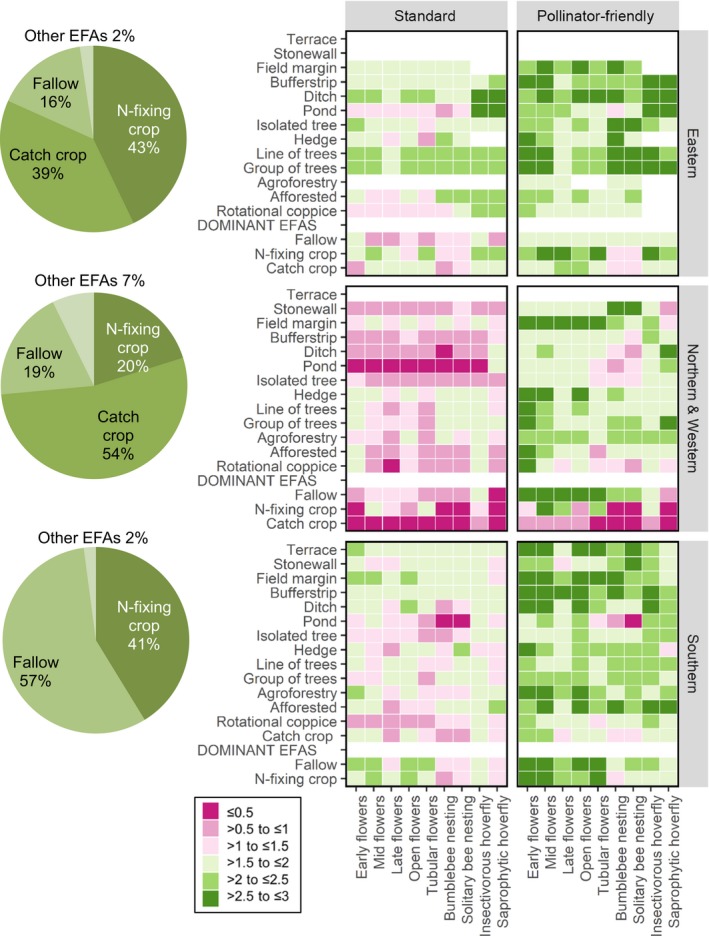
Heat maps illustrating the perceived mean value of Ecological Focus Areas (EFA) options under standard and pollinator‐friendly management for our three European geographical regions. Heat maps are based on the score for each resource type averaged across countries within a region. Missing data represent options with insufficient scores. Pie charts reflect the % area (before applying weighting factors) of EFA options for each region based on the countries in this study (see Table S4 for more detailed information)

For each EFA option, experts scored its potential to provide the selected pollinator resources under standard and under pollinator‐friendly management, with these practices outlined in the evaluation spreadsheet to ensure standardization between evaluators (Table S2). Values were selected from an ordinal scale ranging from 0 (no resource provided) to 3 (high resource availability). To reduce the risk of low confidence in a given score, experts could decline to score where they felt they had insufficient knowledge. Within each geographical region, we aimed to reach a threshold consensus of >66% of scorers selecting the mode. Percentage agreement is the most common definition for consensus, with our 66% criterion being comparable to other studies (i.e. ranging from 50% to 97%) (Diamond et al., 2014).

Following the first round of scoring, mean scores for each region were calculated (i.e. per EFA option, management and resource). These means were included in the second scoring round and experts were invited to revise their initial score in light of the group response, giving justification of their choice. Following calculation of summary statistics from the second scoring round, EFA options not reaching consensus were put forward to a third scoring round, where participants were presented with mean scores derived from round two alongside the rationale/evidence provided by experts in their region. Experts were requested to revise their scores and provide reasoning/evidence behind their chosen score. At this point, deviation between scores was considered to represent true inter‐country variation and/or differences in opinion between experts and scoring was terminated (Appendix S1).

Following evaluations, scores were verified by reviewing comments/evidence provided and validating against information collated in the literature review (Appendix S2). Expert scores typically agreed with the literature, or where significant departures occurred these could generally be attributed to geographical differences in the habitat itself or its management. We note that there was ambiguity in interpretation of the EFA option ‘strips along forest edges’, with some respondents scoring the area adjacent to forest edges (the actual EFA), while others scored the forest edge itself (not an EFA). This EFA option was therefore omitted from the dataset.

### 2.2 Data analyses

For each respondent, three broad resource scores were calculated (i.e. floral, bee nesting and hoverfly larval resources) per EFA option and management. Broad resource scores were calculated as follows: floral resources (mean of early season, mid‐season, late season, open and tubular flowers), bee nesting sites (mean of bumble bee and solitary bee nest sites) and hoverfly larval resources (mean of insectivorous and saprophytic larval resources: Table 1). Although data were collected on an ordinal scale, means were calculated rather than medians to give equal weighting to all resources constituting a broad resource category. The resultant broad resource data allowed the fitting of linear mixed models (LMMs), with EFA option nested in country as random effects to fully capture the hierarchical structure of the data. Preliminary analyses revealed significant three‐ and four‐way interactions between EFA option, management, broad resource type and geographic region (Table S3). To ease interpretation, separate analyses were therefore performed for each of our three geographical regions (i.e. E, N&W and S Europe) and broad resource‐types. Models included EFA option, management and their interaction as fixed factors to enable us to explore whether:

1. Experts perceived current EFA options to differ in their potential to provide resources for pollinators (i.e. fixed effect EFA option).
2. Experts perceived that pollinator‐friendly management promoted pollinator resource value (i.e. fixed effect management).
3. Effects of pollinator‐friendly management on pollinator resource value was perceived to differ among EFA options (i.e. interaction between EFA and management).

LMMs also explored whether EFAs showed seasonal differences in floral resource value. Again a significant three‐way interaction was detected between EFA option, season and geographic region (Table S3). To ease interpretation, separate analyses were therefore conducted for each region under standard management. Here the response variable was the floral resource score with fixed effects EFA option and season (i.e. early, mid and late season), and their interaction. Again, EFA option nested within country were included as random effects.

All analyses were performed in R version 3.5.0 (R Core Team, 2018) using the package nmle (Pinheiro, Bates, DebRoy, & Sarkar, 2018). EFA options were omitted from analyses when scores were obtained from fewer than three countries in a geographic region. In Germany, Greece and Spain, evaluations were provided by more than one expert. To avoid over‐representation bias, scores were averaged over respondents to provide a single score per country, broad resource‐type, EFA and management. Homoscedasticity and normality of residuals were validated by visual inspection of diagnostic plots, with no major departures from normality and equality of variances detected.

## 3 RESULTS

### 3.1 Overall trends

Heat maps of the mean scores achieved by each option highlighted substantial differences in the resources different EFAs provided, and that these changed across geographical regions, seasonally and with management (Figure 2; Table S3). Inter‐country variation was also detected, with hoverfly larval resources in E Europe and nesting resources in E and S Europe showing the greatest variation. Lower inter‐country variation in N&W Europe may reflect the greater availability of research in this region. See Figure S2 for detailed country‐level results for each broad resource category. It is important to note that the Delphi evaluation process may have reduced inter‐country variation within a geographical region due to the process of seeking consensus between scorers (Supporting Information: Delphi Technique).

**Figure 3:**
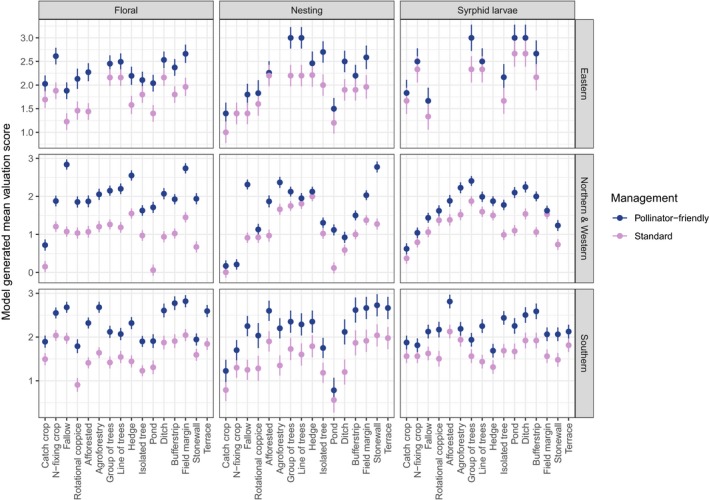
Linear mixed model estimated mean resource scores of different Ecological Focus Areas (EFA) options in the three geographical locations and under standard and pollinator‐friendly management. Error bars indicate ±1 *SE* reflecting variation between countries within a geographical region. Models included EFA, Management and EFA × Management as fixed effects for the following response variables: floral resources, bee nesting sites and hoverfly larval resources. Missing data reflect EFA options with insufficient scores

Heatmaps indicate that under standard management, no single EFA option scored over medium (i.e. >2) for all resources; however, in E Europe, trees in groups/lines only lacked late season floral resources (score = 2). Across EFAs under standard management, perceived resource values tended to be lowest in N&W Europe. This geographical trend was not, however, apparent under pollinator‐friendly management, where N&W resource scores were comparable to other regions.

The bias in EFA uptake towards nitrogen‐fixing crops, fallow land and catch crops (accounting for 97% of total EFA area; European Commission, 2017) is reflected across our three geographical regions (Figure 2; Table S4). Resource scores indicated that even under pollinator‐friendly management, these three EFAs (two EFAs in S Europe where catch crops were not an option) in combination would fail to deliver all necessary resources at good levels (i.e. >2). In E Europe, bee nesting sites received low scores (i.e. ≤2) across these three EFAs, with bumble bee nesting sites also scoring low in the south. Hoverfly larval resources scored low across dominant EFAs in our N&W region, with resources for insectivorous hoverflies also scoring low in S Europe.

### 3.2 EFA options and management across regions

#### 3.2.1 Eastern Europe

In E Europe, EFA options differed in their perceived potential to provide resources (Table 2, Figure 3). Under standard management, floral resource scores were lowest for fallows, ponds, afforested areas and short‐rotation coppices, and highest for ditches, field margins and trees in groups/lines. Alongside catch and nitrogen‐fixing crops, ponds and fallows also received the lowest scores for nesting sites. Afforested areas, while scoring low with respect to floral resources, achieved one of the highest scores for nesting sites. Hoverfly larval resource data were lacking for several EFA options, highlighting a knowledge gap in this region. Experts indicated that ditches and ponds provided most hoverfly larval resources, while fallows, catch crops and isolated trees provided the least.

**Table 2:** Results of linear mixed models examining effects of Ecological Focus Areas (EFA) option, management, and their interaction on pollinator resource value scores

	East		North‐West		South	
	χ^2^ (*df*)	*p*	χ^2^ (*df*)	*p*	χ^2^ (*df*)	*p*
Floral resources						
EFA	45.98 (12)	<.001	159.31 (14)	<.001	89.76 (15)	<.001
Management	68.05 (1)	<.001	192.26 (1)	<.001	121.80 (1)	<.001
EFA × management	16.41 (12)	<.001	90.91 (14)	<.001	16.41 (12)	<.001
Bee nest resources						
EFA	65.54 (12)	<.001	210.23 (14)	<.001	64.82 (15)	<.001
Management	35.49 (1)	<.001	66.62 (1)	<.001	85.53 (1)	<.001
EFA × management	20.40 (12)	.060	107.09 (14)	<.001	15.59 (15)	.410
Syrphid larval resources						
EFA	30.21 (8)	<.001	153.59 (14)	<.001	49.76 (15)	<.001
Management	15.24 (1)	<.001	91.68 (1)	<.001	75.34 (1)	<.001
EFA × management	4.97 (8)	.761	50.99 (14)	<.001	22.66 (15)	.092

Note

Direction and magnitude of effects are presented in Figure 3.

**Figure 4:**
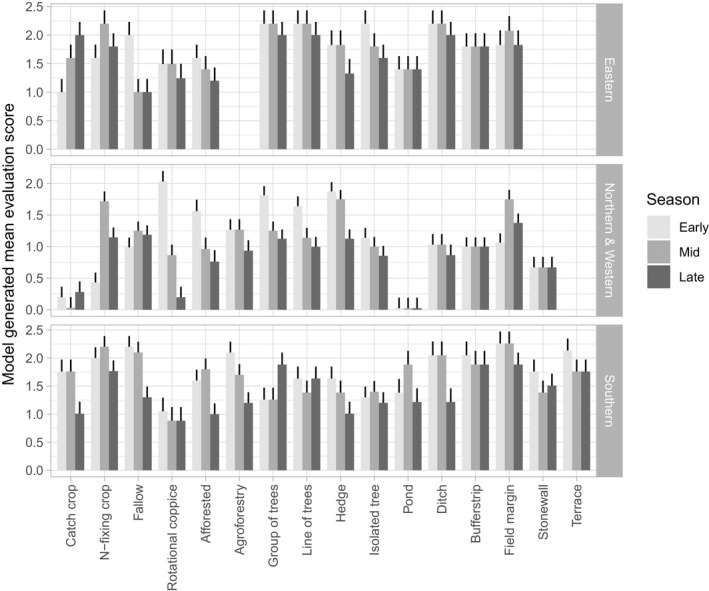
Seasonal variation in floral resource provisioning across different Ecological Focus Areas (EFA) under standard management. Linear mixed model estimated means are presented alongside error bars (±1 *SE*) reflecting variation between countries within a geographical region. Missing data reflect EFA options with insufficient scores

For all EFA options, enhanced pollinator‐friendly management improved the perceived value across resource categories. For hoverfly larval resources and bee nesting sites, pollinator‐friendly management in all EFA options was perceived to increase resources to a similar extent (i.e. no significant EFA × management interaction, Table 2). For floral resources, however, the capacity for management to improve resources differed between EFA options (significant EFA × management interaction; Table 2 and Figure 3). Pollinator‐friendly management had a greater capacity to improve floral resources in afforested areas, fallows, field margins and nitrogen‐fixing crops than in catch crops, isolated trees and trees in a line/group.

#### 3.2.2 Northern and Western Europe

EFA options in N&W Europe showed the greatest differences in pollinator resource scores (Table 2). Under standard management, ponds and catch crops had the lowest floral resource scores, while field margins and hedges had the highest (Figure 3). Ponds and catch crops, together with nitrogen‐fixing crops, also had the lowest scores for bee nesting sites under standard management. Under standard management, nesting site scores were highest for agroforestry, hedges and trees in groups/lines. Under standard management, scores for hoverfly larval resources were lowest for catch crops and highest for trees in groups.

Across the three broad resource options, pollinator‐friendly management improved resource scores, with the magnitude differing between EFA options (Table 2 and Figure 3). Under pollinator‐friendly management, the greatest perceived increase in floral resources occurred in fallows and ponds, while the increase was only marginal in catch crops, isolated trees and nitrogen‐fixing crops. Pollinator‐friendly management did not influence nesting scores of nitrogen‐fixing crops, but did substantially improve nesting scores for fallows and stone walls. Effects of pollinator‐friendly management on hoverfly larval resource scores were most pronounced for ponds and least pronounced for field margins (Figure 3).

#### 3.2.3 Southern Europe

Again, EFA options differed in their potential to provide pollinator resources (Table 2 and Figure 3). Under standard management, fallows, nitrogen‐fixing crops and field margins were evaluated as providing most floral resources, and short‐rotation coppices the least. Bee nesting site scores were highest in terraces and stone walls, and lowest in catch crops and ponds. Hoverfly larval resource scores were highest in afforested areas, agroforestry, buffer strips and ditches, and lowest in hedges and trees in a line.

Across broad resource categories and EFA options, there was an increase in perceived resource quality with pollinator‐friendly management. As in E Europe, effects of management on pollinator resources only varied amongst EFA options for floral resources (significant EFA × management interaction; Table 2). Impacts of management on floral resources were most noticeable in agroforestry and afforested areas, and least pronounced in stone walls and catch crops.

### 3.3 Temporal variation in floral resources across geographical regions

In all three regions, under standard management, seasonal trends in flowering typically differed across EFA options (i.e. significant EFA × season interaction: Table 3 and Figure 4). In N&W Europe, ‘woody habitat’ EFAs (e.g. afforested areas, hedges and trees in lines/groups) were perceived to provide rich, early‐season forage with the resource value typically decreasing as the season progressed. Hedges and afforested areas also scored highly for early‐season forage in S and E Europe, with hedges in E Europe and afforested areas in S Europe continuing to be valuable mid‐season. Fallows scored highly for early‐season resources in S and E Europe, with scores remaining high for this habitat though mid‐season in S Europe.

**Table 3:** Results of linear mixed models examining the effects of Ecological Focus Area (EFA) option, season and their interaction on floral resource value scores

Floral resources (standard management)	East		North‐West		South	
	χ^2^ (*df*)	*p*	χ^2^(*df*)	*p*	χ^2^(*df*)	*p*
EFA	34.89 (12)	<.001	124.94 (14)	<.001	55.45 (15)	<.001
Season	5.47 (2)	.065	19.57 (2)	<.001	29.08 (2)	<.001
EFA × season	62.20 (24)	<.001	173.05 (28)	<.001	61.50 (30)	<.001

Note

Results are based on EFA options under standard management. Direction and magnitude of effects are presented in Figure 4.

Across geographical regions, field margins were perceived to provide high floral resources; however, temporal trends differed. In S and E Europe, field margins were one of the highest scoring EFA options throughout the pollinator activity period (although clear peaks in value were observed early to mid‐season in S Europe). In N&W Europe, however, they lacked early‐season floral resources.

Irrespective of the region, under standard management no EFA had a late‐season floral resource score >2. This was particularly notable in N&W Europe, where no EFA scored >1.5. Late season peaks in floral resources were only detected in catch crops in E Europe and groups of trees in S Europe.

## 4 DISCUSSION

Twenty‐two experts from across Europe evaluated the potential of EFAs (representing a range of habitats and landscape features) under standard and pollinator‐friendly management to support wild pollinators. By considering the seasonal dynamics of floral resources and taxon‐specific life‐cycle requirements, this study expands beyond previous assessments that simply focus on bee floral and nesting resources (Koh et al., 2016; Zulian et al., 2013). With EFA habitats displaying inherent differences in the resources they offer (Baude et al., 2016; Cole, Brocklehurst, Robertson, Harrison, & McCracken, 2017) and these differences varying across Europe, our evaluation provides baseline data to enable Member States to consider pollinator requirements when designing their own choices of options.

### 4.1 Landscape features and floral resources

EFAs varied considerably in their forage value. Across Europe ponds were perceived to provide little in the way of forage while field margins provided particularly rich foraging habitats. Field margins are also perceived as one of the best EFA options for wider biodiversity (Pe’er, Zinngrebe, et al., 2017). The forage value of floristically diverse field margins is well documented (Mendoza‐García, Blanco‐Moreno, Chamorro, José‐María, & Sans, 2018; Sutter, Jeanneret, Bartual, Bocci, & Albrecht, 2017); however, margin mixes are facing criticism for being targeted towards bumble bees, limiting their potential to support other pollinating *taxa* (Campbell, Biesmeijer, Varma, & Wäckers, 2012; Wood, Holland, & Goulson, 2015). Naturally regenerated margins or multi‐functional native species mixes can improve the functional diversity of flowers by increasing the abundance of species with accessible nectaries (e.g. Asteraceae and Apiaceae), favouring a greater diversity of beneficial insects, including parasitic wasps and hoverflies, and thereby improve ecosystem services (pest control; Campbell et al., 2012; Wood et al., 2015).

Pe’er, Zinngrebe, et al. (2017) indicated that nitrogen‐fixing crops provided limited benefits to biodiversity. Our evaluation, however, highlights their potential to provide forage for pollinators, with their protein‐rich pollen being critical for bee reproduction (Scheper et al., 2014). Their forage value, however, varies considerably across Europe, with regional differences driven by both the species grown and the management (e.g. the use of plant protection products and, for fodder crops, the timing and frequency of cutting/grazing). Dominance of field beans, *Vicia faba,* in N&W Europe (particularly in the UK and Netherlands) limits forage value, with deep corolla tubes limiting access by short‐tongued species, and the constrained flowering period reducing the duration of forage availability (Suso et al., 2016). Furthermore, our evaluation was conducted before the use of plant protection products was restricted in EFAs and consequently applications of insecticides and herbicides in *V. faba* were expected to be high, further limiting their value (Underwood & Tucker, 2016). Although worth noting is that this was not the case in the Netherlands where a ban was in place at the time of the evaluation. Within an intensive arable matrix, the value of nitrogen‐fixing crops, particularly forage legumes, in providing protein‐rich pollen should, however, not be underestimated. To capitalize on this potential, cutting/grazing regimes should permit flowering and a diversity of species selected to increase functional diversity, prolonging the flowering period and providing forage for a wider suite of species.

EFA options showed clear seasonal differences in their potential to deliver floral resources, with temporal patterns differing geographically. Field margins were perceived to provide a continuous source of forage in E and S Europe but lacked early season forage in N&W Europe, where woody habitats (e.g. hedgerows and groups of trees) were important in spring instead. With mobile pollinators tracking resources at the landscape scale (Cole et al., 2017; Mandelik, Winfree, Neeson, & Kremen, 2012), habitats that differ in peak flowering time complement each other, stabilizing forage at the landscape scale. For less mobile pollinators (e.g. many species of solitary bees), dispersal between different habitats is less feasible. For such species, the focus should be on improving management in habitats with the potential to provide continuous floral resources (e.g. field margins throughout Europe and fallow land in N&W and S Europe).

Across Europe experts identified a scarcity of late‐season forage, which has been implicated in the decline of late‐active bee species (Scheper et al., 2014). This highlights the importance of management actions that increase late season resources (e.g. including late flowering species in seed mixtures, and staggering and/or more lenient mowing/grazing of nitrogen‐fixing crops).

### 4.2 Landscape features and bee nesting sites

Bees predominantly nest in (semi‐)natural habitats, and the abundance and diversity of bumble bees in farmland indeed increases with proximity to such habitats (Öckinger & Smith, 2007). Bumble bees prefer to nest in areas of dense tussocky grass, embankments and woodland edges, often reusing small mammal nests (Kells & Goulson, 2003). Solitary bees can be broadly divided into ground and cavity‐nesting species, with the availability of bare ground and suitable nesting cavities (e.g. in wood, stonework or pithy plant stems) driving nest site availability (Potts et al., 2005). Habitats perceived to provide the greatest potential for nesting bees (e.g. trees in groups/line and hedgerows in N&W and E Europe and stone walls, afforested areas and terraces in S Europe) offered nesting opportunities for both solitary and bumble bees. In areas where they occur, drystone walls and terraces provide particularly valuable solitary bee nesting sites (Petanidou & Ellis, 1993).

Bees rarely nest in productive crops due to disturbance by infield management (e.g. tillage, harvest, agro‐chemical applications: Scheper et al., 2013), exemplified by the lack of nesting opportunities in catch crops and nitrogen‐fixing crops. With these productive EFA options constituting over 73% of EFAs area, current uptake bias limits the capacity of EFAs to provide bee nesting sites. Habitats typically failed to provide both continuous forage and nesting sites and it is therefore important to consider the spatial configuration of habitats with complementary resources. For example, ensuring flower‐rich habitats such as field margins are in close proximity to good nesting habitats such as hedgerows and stone walls. Such spatial targeting would be particularly beneficial for species with limited dispersal powers (e.g. solitary bees).

### 4.3 Landscape features and hoverfly larval resources

Broadly speaking, hoverfly larval resources were perceived to be most abundant in woody (e.g. agroforestry, afforested areas; Schirmel et al., 2018) and damp habitats (e.g. ditches and ponds), reflecting their diversity of feeding guilds (Jauker, Diekötter, Schwarzbach, & Wolters, 2009; Speight, 2017). Pollinator research is largely biased towards bees and resource requirements of other *taxa* (e.g. hoverflies and parasitic wasps) are often overlooked (Jauker et al., 2009). Our findings indicate that habitats deemed not valuable for bees (i.e. ponds) provide important resources for hoverflies. With hoverflies supplementing pollination in a wide variety of crops (Rader et al., 2016), and many species having predatory larvae that suppress pests (Tschumi et al., 2016), such habitats should not be under‐valued in agroecosystems. Hoverflies are an ecologically diverse group with different species showing habitat specialization towards woody, open and aquatic habitats, highlighting the importance of promoting a diversity of green and blue landscape elements to support them (Schirmel et al., 2018).

### 4.4 Policy implications

With approximately 40% of the EU under agricultural management (European Commission, 2018a), the CAP remains a key policy instrument to tackle pollinator declines. The European Commission proposes to include a pollinator performance indicator within the post‐2020 CAP monitoring framework, highlighting its commitment to conserve pollinators (European Commission, 2018b). The post‐2020 CAP will streamline how it meets environmental objectives under Pillar I (i.e. direct income support) by integrating greening and cross‐compliance regulations through enhanced conditionality (i.e. baseline requirements that must be met to obtain direct income support: European Commission, 2019). Conditionality will see EFAs being replaced by ‘a minimum share of agricultural area devoted to non‐productive features or areas’ under Good Agricultural and Environmental Condition obligations (i.e. GAEC 9). More targeted conservation action will be achieved by continuation of Pillar II rural development vehicles (e.g. agri‐environment and climate measures AECM), and the introduction of eco‐schemes (Pillar I: European Commission, 2019). Eco‐schemes, if implemented effectively, will enable Member States to direct Pillar I funding to address specific regional challenges whilst providing the flexibility to adapt to changing circumstances. Member States will have greater ownership on how they integrate and implement these Green Architecture elements, allowing regional tailoring to local farming systems and conditions. With implementation left largely to the discretion of Member States, however, the CAP post‐2020 lacks clearly defined options and guidelines on the implementation and management of these options. This could weaken environmental outcomes (Pe’er, 2019). Our evaluation provides a baseline to assist Member States consider pollinator requirements when designing their national strategic plans.

Pollinator‐friendly management increases the likelihood that habitats will provide abundant and diverse resources for wild pollinators, potentially also benefiting honeybees (Requier et al., 2015) and other beneficial invertebrates including natural predators (Tschumi et al., 2016). To optimize the benefits derived, the CAP post‐2020 should focus on improving habitat quality, for example incentivizing positive management via result‐based payments. To achieve this, we recommend an effective monitoring framework alongside appropriate target‐orientated indicators (e.g. a specific pollinator indicator in addition to other indicators of ecosystem health such as the EU Butterfly Grassland Indicator; Pe’er et al., 2019). Even under pollinator‐friendly management, however, only ditches in E Europe and buffer strips (especially of perennial shrubs) in S Europe were perceived to provide all necessary resources at sufficient quantities. This highlights that measures to simply improve habitat quality may not be sufficient. Furthermore, as a result of current uptake bias towards nitrogen‐fixing crops, fallow land and catch crops (i.e. 97% of EFA area: European Commission, 2017) experts perceived shortages in bee nesting sites, late season forage and hoverfly larval resources. Restricting eligible landscape elements to non‐productive features/areas could address this uptake bias; however, this clearly depends on implementation.

To safeguard pollinators in agroecosystems, the post‐2020 CAP needs to progress beyond simply improving habitat quantity to explore options that increase habitat quality, connectivity and complementarity to ensure that pollinators have access to all necessary resources in sufficient quantities. Fundamental to achieving this is a better understanding of the level of resources required to sustain healthy populations, and also the level of resources currently present in a landscape. Robust scientific data in this field is, however, largely lacking, highlighting the need for targeted research in this area. While our evaluation provides a comprehensive baseline evaluation of the resource potential of non‐productive habitats across Europe, we recommend Member States work directly with pollinator experts in their region to ensure that pollinator requirements are taken into account. In addition, an effective participatory monitoring framework, backed with scientific knowledge, will help to keep track of effectiveness and identify where refinement is required to improve outcomes.

Our evaluation indicates that as a result of the inherent capacity of habitats to provide different resources, inadequate management and uptake bias, EFAs are largely failing to deliver all necessary pollinator resources at sufficient quantities in European agricultural landscapes. Targeted pollinator‐friendly management, can help address this shortfall in resources. Beyond this, the post‐2020 CAP could deliver further benefits through landscape‐level initiatives that support combinations of options targeted to provide complementary pollinator resources. Effective delivery would require the integration of Pillar I (conditionality and eco‐schemes) and Pillar II (AECM and support for organic/high nature value farming) vehicles with means of incentiviszng collaboration between farmers and other stakeholders to spatially target measures (Bartomeus & Dicks, 2019). For example, eco‐schemes and AECM could be regionally targeted to complement habitats delivered under conditionality, thus fulfilling shortfalls in resources. A more joined‐up approach to the implementation of the post‐2020 CAP will not only benefit pollinators but also wider biodiversity (Nilsson et al., 2019; Pe’er et al., 2019).

As we approach the CAP post‐2020, our European‐scale evaluation highlights that to effectively conserve pollinators and help protect pollination services, there is a need to improve habitat quality and exploit habitat complementarity. Through adopting an integrated approach to Green Architecture, it is our vision for the post‐2020 CAP to deliver a diversity of interconnected, high‐quality habitats tailored across Europe to local farming systems and conditions. Such pollinator‐friendly landscapes would not only help conserve pollinators within intensive agricultural matrices, but also help connect isolated areas of high nature value farmland and protected sites, often critical for species of conservation concern.

## AUTHORS’ CONTRIBUTIONS

L.J.C., L.V.D., D.K., J.C.S. and S.G.P. conceived the idea and designed methodology. L.J.C. and J.S. formulated and analysed the data. L.J.C., D.K. and J.S. wrote the initial draft. All authors contributed to the writing the manuscript, the evaluation process/formulation of pollinator‐friendly management options and gave approval for final publication.

## Data Availability

Data are available via the Dryad Digital Repository https://doi.org/10.5061/dryad.ht76hdrbn (Cole et al., 2020).
