# Supplementary figures and images for: A critical analysis of the potential for EU Common Agricultural Policy measures to support wild pollinators on farmland
Source: J Appl Ecol. 2020 Feb 16;57(4):681–94. doi: 10.1111/1365-2664.13572 (PMC7188321; doi:10.1111/1365-2664.13572)

**Figure S1. Overview schematic of the Delphi evaluation process adopted in this study.**

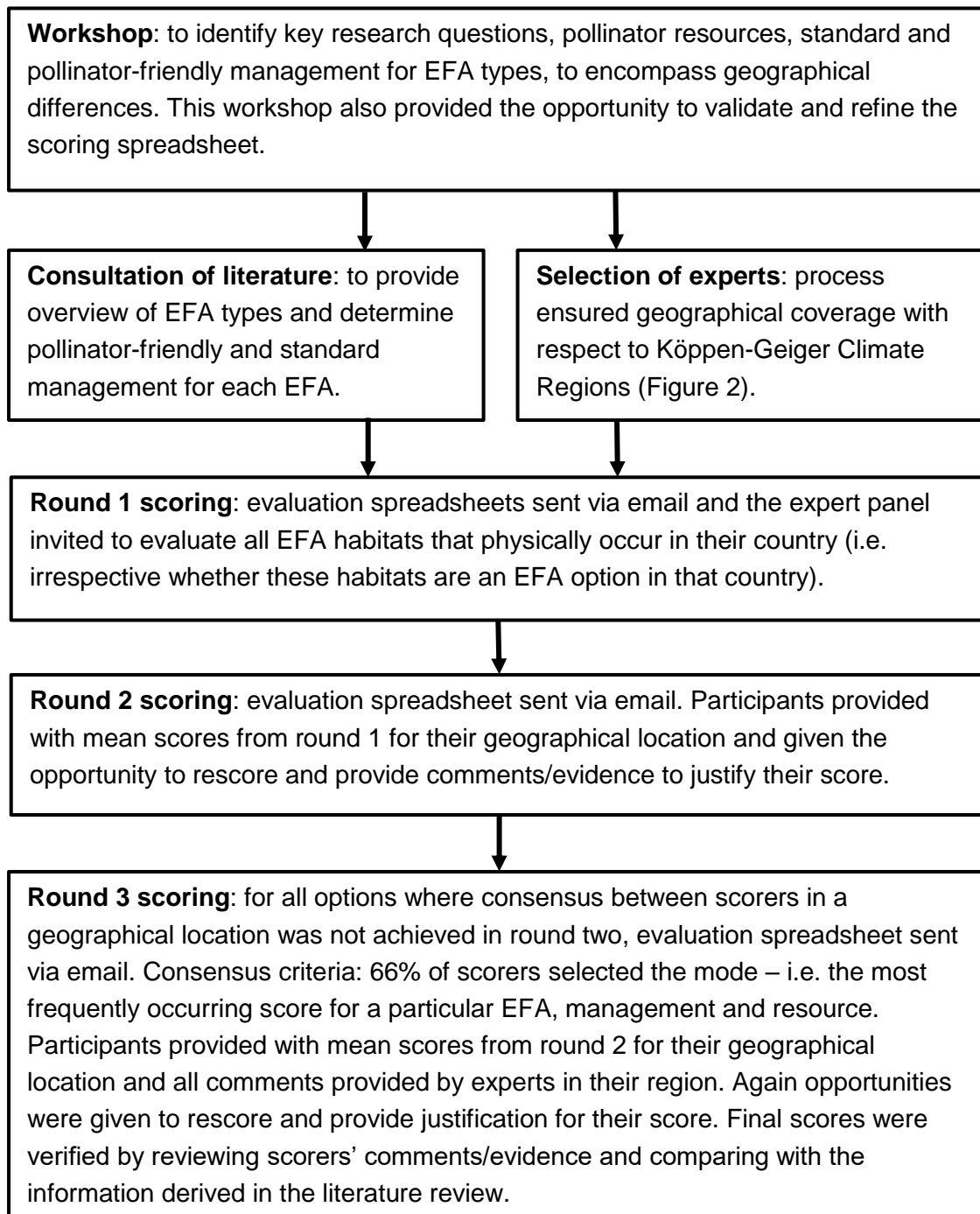

Supplement: Supplementary file 1 [file JPE-57-681-s001.pdf]
