# Supplementary material for: A critical analysis of the potential for EU Common Agricultural Policy measures to support wild pollinators on farmland
Source: J Appl Ecol. 2020 Feb 16;57(4):681–94. doi: 10.1111/1365-2664.13572 (PMC7188321; doi:10.1111/1365-2664.13572)

Figure S2 a. Broad resource scores derived for each EFA option for the five countries representing our Eastern European region. Resource scores are provided under standard and pollinator-friendly management. Missing data reflects EFA options that were not scored.

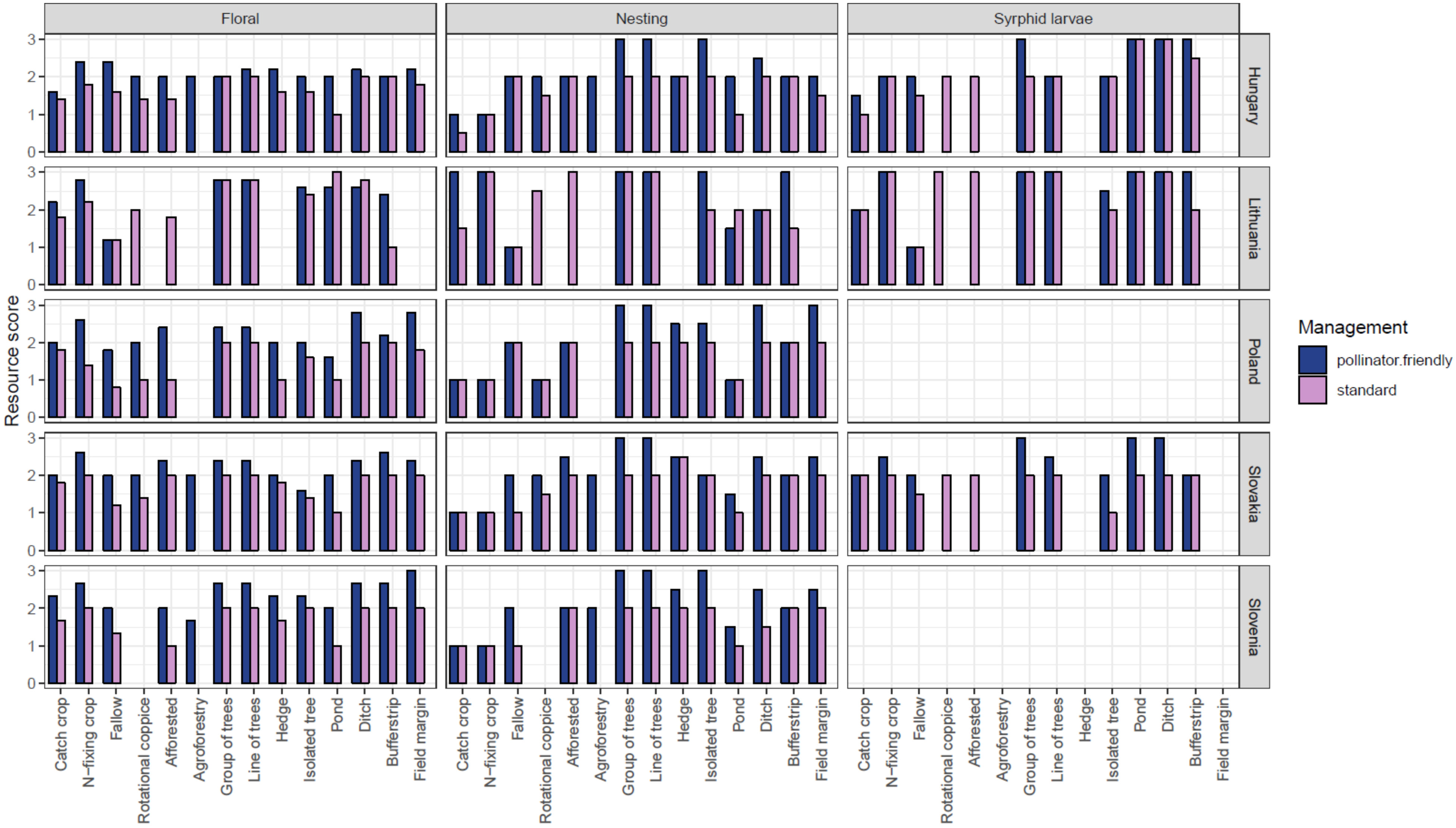

Figure S2b. Broad resource scores derived for each EFA option for the eight countries representing our Northern & Western European region. Scores are provided under standard and pollinator-friendly management. For countries with more than one scorer, the mean resource score is calculated across scorers and the associated standard error provided. Missing data reflect EFA options that were not scored.

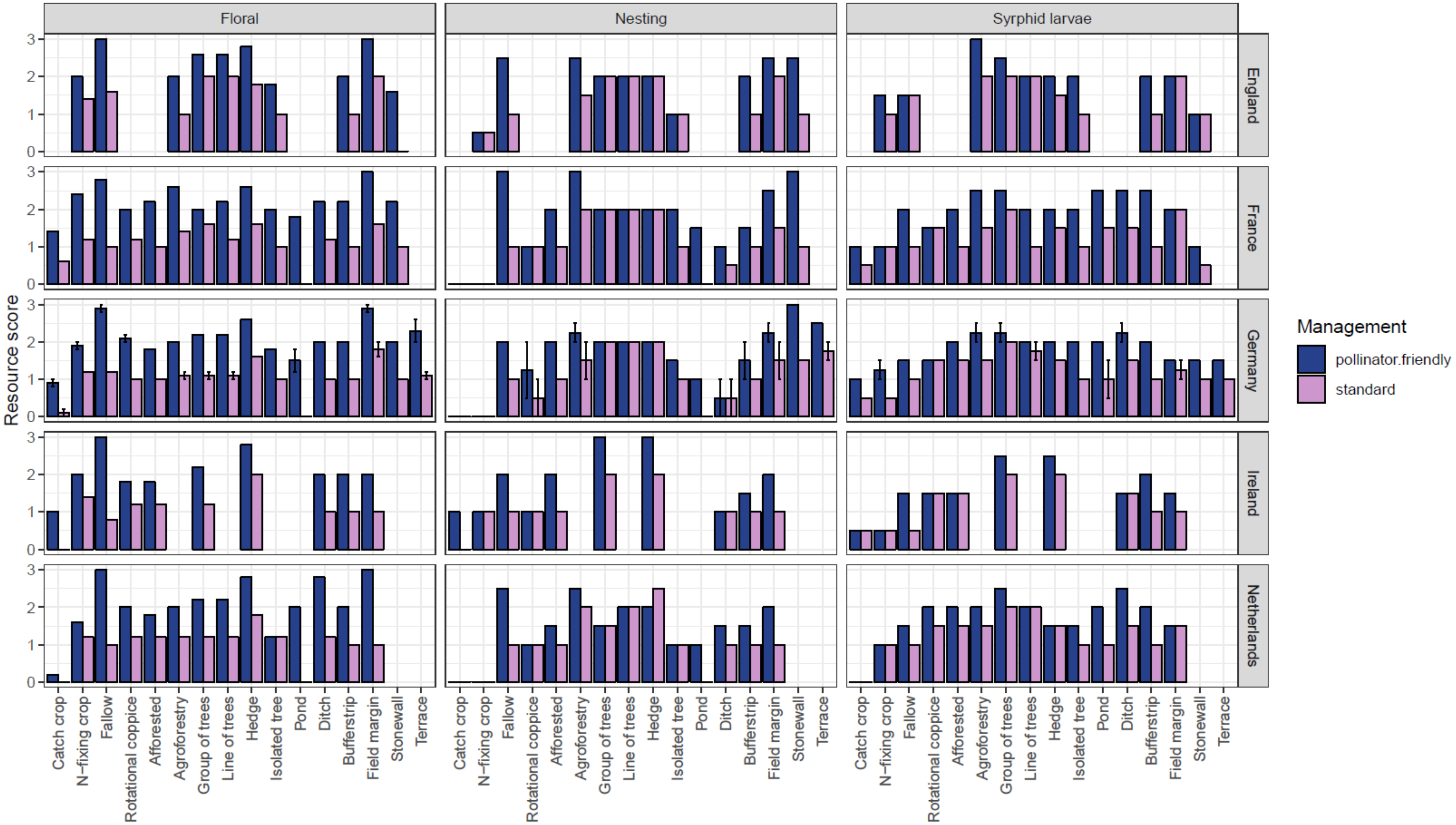

Figure S2b continued.

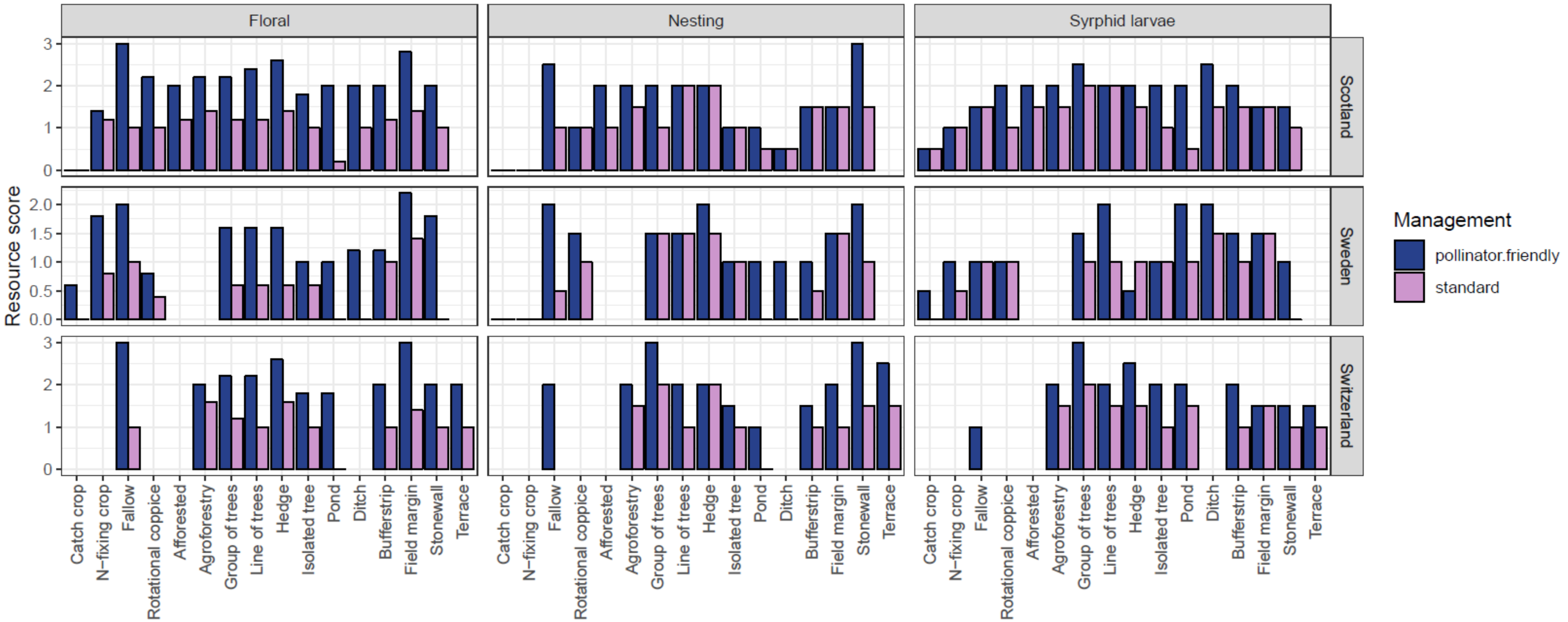

Figure S2c. Broad resource scores derived for each EFA option for the five countries representing our Southern European region. Resource scores are provided under standard and pollinator-friendly management. For countries with more than one scorer, the mean resource score is calculated across scorers and the associated standard error provided. Missing data reflect EFA options that were not scored.

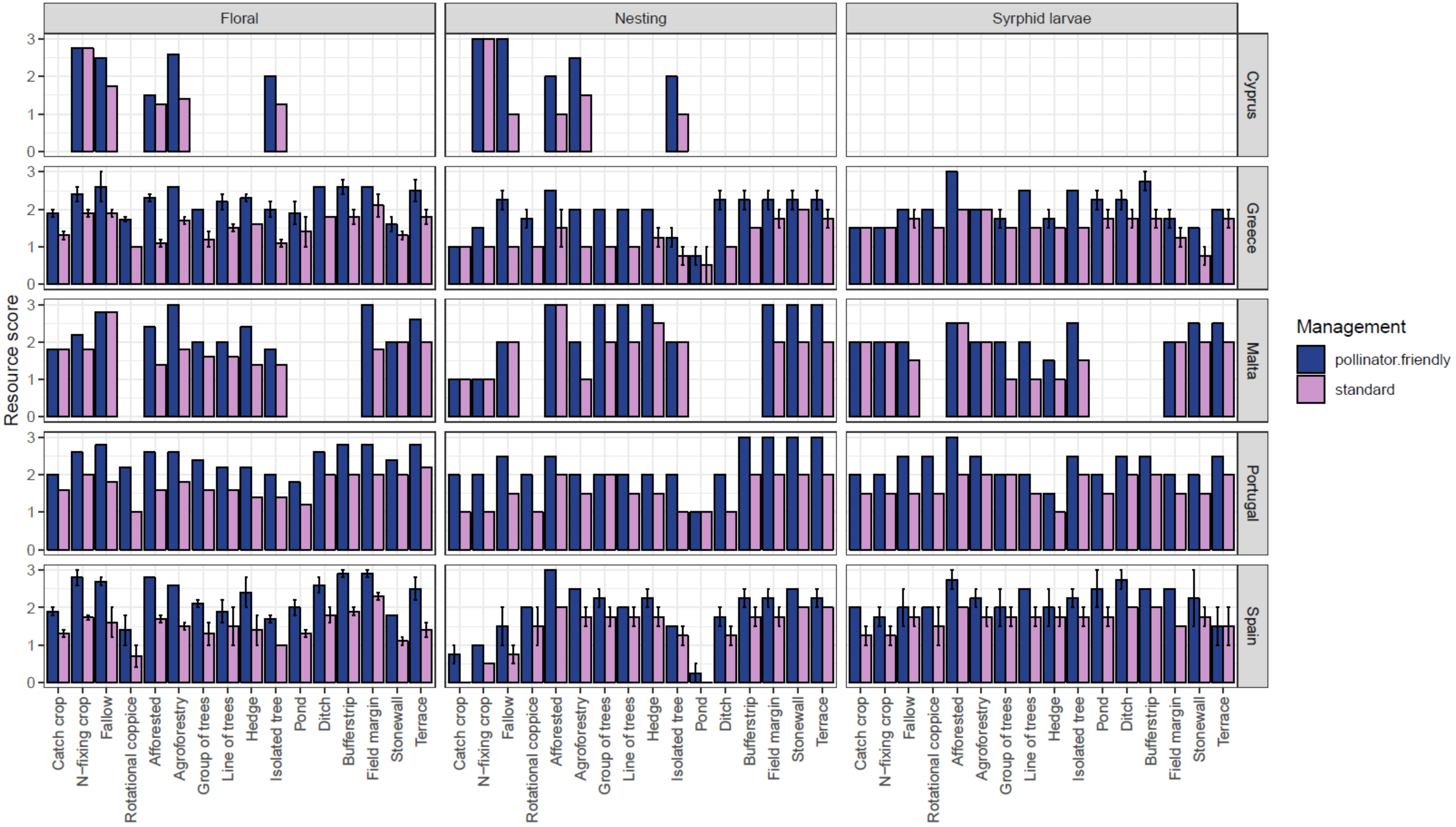

Supplement: Supplementary file 2 [file JPE-57-681-s002.pdf]
