# Supplementary material for: A critical analysis of the potential for EU Common Agricultural Policy measures to support wild pollinators on farmland
Source: J Appl Ecol. 2020 Feb 16;57(4):681–94. doi: 10.1111/1365-2664.13572 (PMC7188321; doi:10.1111/1365-2664.13572)
Supplement: Supplementary file 3 [file JPE-57-681-s003.pdf]

Table S1. Overview of Ecological Focus Areas, including information on the weighting factor and number of countries taking up the option (European Commission, 2017).

Ecological Focus Area	***Brief description***	***Weight-ing***	***No. of countries***
Nitrogen fixing crops	Sowing plants belonging to the family Fabaceae (e.g. lucerne, field beans and clover)	0.7	31
Catch crops/ Green cover	Sowing selected species (e.g. mustard, phacelia, fodder radish) post-harvest or under-sowing with grass	0.3	21
Afforested areas	Newly converted areas of arable land into woodland (i.e. tree plantation)	1	15
Agroforestry	Integration of trees within a productive agricultural cropping or livestock system	1	11
Buffer strips	Unproductive arable field margins adjacent to watercourses (or in some member states on upper slopes)	1.5	19
Forest edges – with production	Productive arable field margins adjacent to a forest edge managed with reduced agro-chemical inputs Ecotone habitat	0.3	6
Forest edges - without production	Unproductive arable field marg ins adjacent to a forest edge. Ecotone habitat	1.5	10
Land lying fallow	Previous arable land without production (i.e. no grazing/cropping). Land may be sown or left to naturally regenerate	1	30
Short Rotation Coppice	Densely planted, fast growing varieties of trees (e.g. *Salix* and *Populus* spp.) typically harvested after a short period of time (e.g. 4 years) as an energy crop	0.3	22
Terraces	Man-made levelled land strips permitting/ facilitating cultivation on slope terrains, supported mostly by drystone walls and in few cases by natural low-scrub vegetation	1	7
Traditional stone walls	Walls constructed of natural drystone that are typically associated with field boundaries	1	8
**Landscape features:**			
Ditches	Ditches with a maximum width of 6 m and associated vegetated ditch banks. Typically boundary feature for the purpose of irrigation or drainage	2	16
Field margins	Vegetated field margins (1-10 m wide) with no agricultural production. Field margins may be sown or left to regenerate naturally	1.5	17
Hedges	Strip of shrubs/trees adjacent to arable land, with a maximum width of 10 m	2	16
Isolated trees	Single tree located within a field typically with minimum crown diameter of 4 m	1.5	13
Trees in groups	Group of trees connected by overlapping crown cover and field copses with a maximum area of 0.3 ha	1.5	18
Trees in line	Line of woody trees of more than 20 m in length. Minimum crown diameter of 4 m with gaps between tree crowns not exceeding 5 m	2	16
Ponds	Natural freshwater pond with an area < 0.1 ha	1.5	13
