# Supplementary material for: A critical analysis of the potential for EU Common Agricultural Policy measures to support wild pollinators on farmland
Source: J Appl Ecol. 2020 Feb 16;57(4):681–94. doi: 10.1111/1365-2664.13572 (PMC7188321; doi:10.1111/1365-2664.13572)
Supplement: Supplementary file 4 [file JPE-57-681-s004.docx]

**Table S2.** Overview of both standard (i.e. typical management across EU countries and regions) and pollinator friendly management (i.e. enhanced management specifically targeted to promote insect pollinators) of Ecological Focus Areas.

| ***Ecological Focus Area*** | ***Standard management*** | ***Pollinator friendly management*** |
| --- | --- | --- |
| Nitrogen fixing crops | • Minimum area in some countries.  • Crop has to be present for a certain amount of time in some countries.  • Specific choices of species (differs greatly across countries). | • Use nectar and pollen-rich species (e.g. lupins, clover)  • Include more than one species to increase continuity/ diversity of floral resources.  • Restrict agro-chemical inputs*. Use Integrated Pest Management.  • Avoid planting flowering crops following crops receiving neonicotinoids.  • Allow plants to flower. |
| Catch crops/ Green cover | • Catch crop sown after the main crop.  • Under-sowing grass in the main crop.  • Catch crop should be established for a minimum period and sowing dates are specified by country.  • Catch crops are typically in situ during winter and ploughed into the soil in spring.  • Mixtures with specified plant species and seed density are defined by some countries. | The following management options are more pertinent to South Europe where flowering can occur prior to spring ploughing:  • Allow plants to flower (e.g. delay ploughing so catch crop can provide early season resources).  • Use nectar and pollen-rich species (e.g. clover, *Phacelia*) and include more than one species to increase continuity/diversity of floral resources.  • Avoid using insecticide seed coating and plant protection products during the catch crop presence in the field.  • Avoid putting flowering catch crops in after a neonicotinoid treated crop. |
| Afforested areas | • Use native species well-adapted to local edapho-climatic conditions (Portugal); spot treatment of invasive non-native species allowed (UK-Wales).  • Mild exploitation for food/raw-material production allowed (Portugal).  • Eligible species are alder, silver birch, European ash, sweet chestnut, willow, poplar, hazel, lime and sycamore (UK-Wales). | • Select nectar and pollen-rich (non-toxic) woody species with diverse characteristics (e.g. flower shape, flowering period).  • Encourage growth of understory and perennial herbs (e.g. via late season mowing/ grazing, reduced planting density and selecting trees that facilitate understory development).  • If invasive plants/injurious weeds must be controlled, use only targeted mechanical or spot treatments.  • Do not remove dead wood, stones.  • The shape of the proposed woodland should aim to increase the edge to interior ratio (e.g. shapes with complex geometry and a greater perimeter to area ratio are preferred).  • Maintain patches of bare ground and a balance of annual and perennial plant species. |
| Agroforestry | • Fertiliser and pesticide applications are typically restricted and problematic plants treated by spot treatment of herbicides.  • In extensively managed orchard meadows:  at least one cut/ year no earlier than 15 June in the lowlands, or 15 July in the mountain zone; cut plant material (except dead wood) must be removed; grazing allowed only in autumn (1 September to 30 November); no mulching allowed. Maintenance for at least 8 years.  • In extensively managed orchard pastures: regular occurrence of a set of indicator plant species; Maintenance for at least 8 years.  • Specification of tree height, density and spacing, crown diameter | • Select nectar and pollen-rich (non-toxic) woody species with diverse characteristics (e.g. flower shape, flowering period).  • Encourage growth of understory and perennial herbs (e.g. via late season mowing/ grazing, reduced planting density and selecting tree species that facilitate understory development).  • Do not remove dead wood, stones.  • Maintain patches of bare ground and a balance of annual and perennial plant species  • Restrict agro-chemical inputs. Use Integrated Pest Management.  • If invasive plants/injurious weeds must be controlled, use only targeted mechanical or spot treatments. |

**Table S2.** Continued

| ***Ecological Focus Area*** | ***Standard management*** | ***Pollinator friendly management*** | |
| --- | --- | --- | --- |
| Buffer strips | • Buffer strips may be established adjacent to watercourses (i.e. riparian buffer strips) or in some Member States on upper slopes to intercept run-off (i.e. non-riparian buffer strips).  • In line with cross-compliance regulations the minimum width for buffer strips is 1 – 2 m with member states having the option to increase this width.  • Grazing and/or cutting is permitted (i.e. but not required) by all member states.  • Control of injurious weeds permitted by spot treatment of herbicides. | • Introduce low level disturbance (e.g. via restricted grazing or mowing) to encourage floristic diversity. Frequency is dependent on Member State and site conditions.  • Stagger mowing to avoid seasonal gaps in floral resources.  • Place buffers adjacent to watercourses rather than on upper slopes to spatially target botanically diverse locations.  • Minimum width of 6 m to reduce disturbance associated with adjacent agricultural fields.  • Protect from field management practices to avoid damage (e.g. compaction, spray drift, ploughing).  • Avoid dominance by coarse vegetation (e.g. reeds and *Phalaris canariensis*). |  |
| Forest edges – with or without production | • Grazing and cutting are permitted.  • Minimum widths of 3 or 6 m are set in some countries, but for many the minimum width is 1 m.  • Ploughing is permitted in forest edges with production | • Facilitate the natural regeneration of nectar and pollen-rich (non-toxic) woody species with diverse characteristics (e.g. flower shape, flowering period).  • Restrict agro-chemical inputs. Use Integrated Pest Management (of particular relevance to forest edges with production).  • Encourage herbaceous/scrub at forest edge to create a more graduated ecotone (i.e. soft edge).  • Select forest edges where the aspect provides favourable conditions for pollinators (i.e. south facing for sun and warmth in northern countries and north facing for shade in southern countries).  • Do not remove dead wood, stones.  • Maintain patches of bare ground and a balance of annual and perennial plant species.  • If invasive plants/injurious weeds must be controlled, use only targeted mechanical or spot treatments |  |
| Land lying fallow | • No cultivation of crops during a minimum period (e.g. during the first half of the year), but generally no specifications about no. of years/cultivation cycles  • Land must be “kept in good condition”: mowing, grazing, mechanical or chemical weed control, fertilizer application may therefore be, under restrictions, allowed in some countries, while not allowed in others; also variable prescriptions regarding timing of management (e.g. cutting) and removal of biomass. | • Retain fallow for more than one year to encourage both annual and perennial forbs. Consider allowing longer term succession in which woody species are allowed to grow.  • Introduce low level disturbance (e.g. via restricted grazing or mowing) to encourage floristic diversity. Frequency dependent on Member State and site conditions.  • Maintain patches of bare ground and a balance of annual and perennial plant species.  • Stagger mowing to create a diversity of successional stages and/or to avoid seasonal gaps in floral resources.  • Avoid pesticide/fertilizer use.  • If invasive plants/injurious weeds must be controlled, use only targeted mechanical or spot treatments.  • Spatially target botanically diverse locations (e.g. areas of previous low intensity management).  • If naturally regenerated vegetation provides poor floral resources for pollinators encourage botanical diversity via sowing of wildflower mixtures. Select resource-rich species with diverse characteristics (e.g. flower shape, flowering period). |  |

**Table S2.** Continued

| ***Ecological Focus Area*** | ***Standard management*** | ***Pollinator friendly management*** | |
| --- | --- | --- | --- |
| Short Rotation Coppice | • Limitations on use of mineral fertilisers and plant protection products (in most countries).  • Harvesting after 4 years. | Select nectar and pollen-rich (non-toxic) woody species  • Include more than one species to increase continuity/diversity of floral resources.  • Stagger harvesting to ensure some areas of the SRC flower every year (i.e. to provide continuity of resources across years).  • Encourage growth of understory and perennial herbs (e.g. via late season mowing/grazing, reducing planting density and selecting tree species that facilitate understory development).  • Restrict agro-chemical inputs. Use Integrated Pest Management.  • If invasive plants/injurious weeds must be controlled, use only targeted mechanical or spot treatments. |  |
| Terraces | • Retention of rock terraces (cross compliance)  • Minimum terrace dimensions (Hungary): minimum 1 meter high, 2 meter wide, 10 meter long  • Protection of land terraces in vineyards obligatory (Hungary) | • Stonewall maintenance should avoid mortar or cement.  • Restrict agro-chemical inputs. Use Integrated Pest Management.  • Cultivate terraces to maintain patches of bare ground and a balance of annual and perennial plant species.  • If invasive plants/injurious weeds must be controlled, use only targeted mechanical or spot treatments.  • Introduce low level disturbance (e.g. via restricted grazing or mowing). Frequency dependent on Member State and site conditions. |  |
| Traditional stone walls | • Traditional building, without use of mortar or cement  • Minimum and maximum dimensions (e.g. with respect to height, width and length). | • Maintain a vegetated buffer (minimum width 2m) adjacent to feature where ploughing and use of agro-chemicals is not permitted.  • In buffer introduce low level disturbance (e.g. via restricted grazing or mowing and removal of cuttings). Frequency dependent on Member State and site conditions.  • Maintain patches of bare ground and a balance of annual and perennial plant species.  • Stonewall maintenance should avoid use of mortar or cement.  • Wider walls have greater potential for small mammals and thus provide disused cavities to provide bumblebee nesting sites. |  |

**Table S2.** Continued

| ***Ecological Focus Area*** | ***Standard management*** | | ***Pollinator friendly management*** | |
| --- | --- | --- | --- | --- |
| **Landscape features:** | | |  |  |
| Ditches | | • In most parts of North-western and Central Europe ditch banks are cut by flail mower and cuttings are left on the banks. | • Introduce low level disturbance (e.g. via restricted grazing or mowing) to encourage floristic diversity. Frequency dependent on Member State and site conditions.  • Maintain a vegetated buffer (minimum width 2 m) adjacent to feature where ploughing and use of agro-chemicals are not permitted.  • Protect from field management practices to avoid damage (e.g. compaction, spray drift, ploughing).  • If invasive plants/injurious weeds must be controlled, use only targeted spot treatments.  • Avoid pesticide/fertilizer use.  • Avoid dominance by coarse vegetation (e.g. reeds and *Phalaris canariensis*) |  |
| Field  margins | | • Minimum width of 1-20 m  • No agricultural production allowed  • In some Member States, fertilizer is allowed for establishment of seed mixes (Scotland)  • In some Member States, injurious, invasive and other weeds should be controlled by spot spraying of herbicide (Scotland) or hand weeding (Hungary, Poland)  • Other pesticides (including insecticides) are not allowed | • If naturally regenerated vegetation provides poor floral resources for pollinators encourage botanical diversity via sowing of wildflower mixtures. Select nectar and pollen-rich species with diverse characteristics (e.g. flower shape, flowering period).  • Introduce low level disturbance (e.g. via restricted grazing or mowing) to encourage floristic diversity. Frequency dependent on Member State and site conditions.  • Stagger mowing to avoid seasonal gaps in floral resources.  • If invasive plants/injurious weeds must be controlled, use only targeted mechanical or spot treatments.  • Spatially target botanically diverse locations (e.g. adjacent to watercourses, areas of previous low intensity management).  • Minimum width of 6m to reduce disturbance associated with adjacent agricultural field  • Protect from field management practices to avoid damage (e.g. compaction, spray drift, ploughing). | |
| Hedges | | • Maximum width in some countries (up to 10m in Estonia and Hungary).  • Vegetation should be bushes and trees (Estonia) or woody material (UK-NI and HU)  • Cross compliance rules also apply which include not cutting between 1 March and 31 August, although there are exemptions. | • Select nectar and pollen-rich (non-toxic) woody species with diverse characteristics (e.g. flower shape, flowering period).  • Maintain a vegetated buffer (minimum width 2 m) adjacent to feature where ploughing and use of agro-chemicals is not permitted.  • Hedge cutting interval should allow shrubs/plants to flower (i.e. at least two years between cuts) and cutting should be staggered within a farm to ensure some hedgerows flower every year.  • Protect from field management practices to avoid damage (e.g. compaction, spray drift, ploughing).  • If invasive plants/injurious weeds must be controlled, use only targeted mechanical or spot treatments. | |

**Table S2.** Continued

| ***Ecological Focus Area*** | ***Standard management*** | | ***Pollinator friendly management*** | |
| --- | --- | --- | --- | --- |
|  | |  | |  |
| Isolated    Trees | | • Minimum crown diameter of 4m.  • No pesticide or fertiliser applied within 3 m radius of the trees.  • Cannot be cut. | | • Encourage growth of understory and perennial herbs (e.g. via late season mowing/grazing).  • Maintain a vegetated buffer (minimum width 5 m) adjacent to feature where ploughing and the use of agrochemicals is not permitted.  • Do not remove dead wood, stones.  • If invasive plants/injurious weeds must be controlled in the vegetated buffer, use only targeted mechanical or spot treatments. |
| Trees in   Groups | | • Perished trees do not require replacement.  • Management, such as pruning, during dormancy applied to no more than a third of the total area.  • No pesticide or fertiliser applied within 3 m radius of the trees.  • Soil cultivation prohibited, except for grassland reconstruction works. | | • Select nectar and pollen-rich (non-toxic) woody species with diverse characteristics (e.g. flower shape, flowering period).  • Maintain a vegetated buffer (minimum width 5m) adjacent to feature where ploughing and use of agro-chemicals is not permitted.  • Encourage growth of understory and perennial herbs (e.g. via late season mowing/grazing, reduced planting density and selecting trees that facilitate understory development).  • Encourage herbaceous/scrub at woodland edge to create a more graduated ecotone (i.e. soft edge).  • Do not remove dead wood, stones.  • Maintain patches of bare ground and a balance of annual and perennial plant species.  • If invasive plants/injurious weeds must be controlled, use only targeted mechanical or spot treatments. |
| Trees in line | | • Perished trees must be replaced.  • Minimum crown diameter of 4m, minimum separation of tree crowns 5m, minimum length of the line of trees 20-25m and minimum area 0.1 ha (based on crown diameter).  • Management of cutting regime, such as pruning, during dormancy applied to no more than a third of the total area.  • No pesticide or fertiliser applied within 3 m radius of the trees. | | • Select nectar and pollen-rich (non-toxic) woody species with diverse characteristics (e.g. flower shape, flowering period).  • Introduce low level disturbance (e.g. via restricted grazing or mowing) to encourage floristic diversity. Frequency dependent on Member State and site conditions.  • Do not remove dead wood, stones.  • Maintain patches of bare ground and a balance of annual and perennial plant species.  • If invasive plants/injurious weeds must be controlled, use only targeted mechanical or spot treatments.  • Maintain a vegetated buffer (minimum width 3 m) adjacent to feature where ploughing and use of agro-chemicals is not permitted.  • Ensure aspects that provide favourable conditions for pollinators are protected from field management practices (i.e. south facing for sun and warmth in northern countries and north facing for shade in southern countries) |

**Table S2.** Continued

| ***Ecological Focus Area*** | ***Standard management*** | ***Pollinator friendly management*** |
| --- | --- | --- |
| Ponds | • Maximum 0.1 ha (minimum 0.01 ha in Belgium and Lithuania)  • Has to be maintained in Hungary | • Maintain a vegetated buffer (minimum width 5 m) adjacent to feature where ploughing and use of agro-chemicals is not permitted.  • In the buffer introduce low level disturbance (e.g. via restricted grazing or mowing) to encourage floristic diversity. Frequency dependent on Member State and site conditions.  • If invasive plants/injurious weeds must be controlled, use only targeted mechanical or spot treatments.  • Avoid dominance by coarse vegetation (e.g. reeds and *Phalaris canariensis*)  • Protect from field management practices to avoid damage (e.g. compaction, spray drift, ploughing). |

*Outlining standard and pollinator-friendly management options for EFAs and initial scoring was conducted prior to banning of plant protection products in 2018

Correction note: Table S2 duplicate entry for Ditches removed 18/02/2020
