# Supplementary material for: A critical analysis of the potential for EU Common Agricultural Policy measures to support wild pollinators on farmland
Source: J Appl Ecol. 2020 Feb 16;57(4):681–94. doi: 10.1111/1365-2664.13572 (PMC7188321; doi:10.1111/1365-2664.13572)
Supplement: Supplementary file 5 [file JPE-57-681-s005.pdf]

Table S3. Estimates of linear mixed model testing four-way interaction effects of EFA type, resource type, management, and geographical region on pollinator resource value scores. Results of separate models testing three-way interaction effects of EFA type, resource type, and geographical region, per management type are also shown. Finally estimates of linear mixed models testing three-way interaction effects of EFA type, season and geographical location on the response variable floral resource scores are provided.

**Response variable and mode**	χ^2	**Probability**
Response variable: Resource score		
Four-way interaction		
EFA x Resource x Management x Geographical region	$57.69_{(32)}$	<0.005
Three-way interaction: standard management		
EFA x Resource x Geographical region	$56.38_{(32)}$	<0.005
Three-way interaction: pollinator-friendly management		
EFA x Resource x Geographical region	$85.97_{(32)}$	<0.001
Response variable: Floral resource score		
Three-way interaction: standard management		
EFA x Season x Geographical region	$175.56_{(48)}$	<0.001
