# Supplementary material for: A critical analysis of the potential for EU Common Agricultural Policy measures to support wild pollinators on farmland
Source: J Appl Ecol. 2020 Feb 16;57(4):681–94. doi: 10.1111/1365-2664.13572 (PMC7188321; doi:10.1111/1365-2664.13572)
Supplement: Supplementary file 6 [file JPE-57-681-s006.pdf]

Table S4. Uptake of EFA options for countries representing our three geographical regions. The total land area (i.e. before the application of weighting factors) of each EFA across countries in a region is provided (in thousands ha) alongside the percentage based on the total EFA land area in that region. Data from France and Scotland were not available and areas of landscape features are combined (data for 2015 sourced from European Commission 2017).

Ecological focus Area option	Eastern	Northern & Western	Southern
Nitrogen-fixing crops	764.48 (42.89%)	444.6 (20.14%)	781.55 (41.25%)
Catch crops	691.79 (38.82%)	1,178.53 (53.39%)	0 (0%)
Land lying fallow	284.58 (15.97%)	423.72 (19.19%)	1,073.37 (56.66%)
**Total Area**	**1,740.85 (97.68%)**	**2,046.85 (92.72%)**	**1,854.92 (97.91%)**
Landscape features	15.25 (0.86%)	119.52 (5.41%)	0.42 (0.02%)
Afforested areas	9.91 (0.56%)	2.68 (0.12%)	38.93 (2.05%)
Forest strips	8.47 (0.48%)	0.61 (0.03%)	0 (0%)
Buffer strips	4.96 (0.28%)	31.45 (1.42%)	0.26 (0.01%)
Short rotational coppice	2.77 (0.16%)	6.45 (0.29%)	0 (0%)
Terraces	0.02 (0.001%)	0 (0%)	0 (0%)
Agroforestry	0 (0%)	0.01 (0.0005%)	0 (0%)
**Total Area**	**41.38 (2.32%)**	**160.72 (7.28%)**	**39.61 (2.09%)**
