# Supplementary material for: A critical analysis of the potential for EU Common Agricultural Policy measures to support wild pollinators on farmland
Source: J Appl Ecol. 2020 Feb 16;57(4):681–94. doi: 10.1111/1365-2664.13572 (PMC7188321; doi:10.1111/1365-2664.13572)
Supplement: Supplementary file 7 [file JPE-57-681-s007.pdf]

## Appendix S1: Delphi Technique

The Delphi Technique is a structured method of expert/participant evaluation, well established in the fields of medicine, policy and economics. The Delphi Technique is a powerful tool to seek consensus where comprehensive data is lacking, or data conflicting, and its potential value to ecology and conservation is becoming increasingly recognised (Mukherjee et al., 2015). When compared to other group based elicitation processes the Delphi process ensures anonymity between experts and thus is perceived to be less influenced by social pressure that can inforce consensus (e.g. where peer pressure or over assertive participants may force consensus, halo-effect). That said the Delphi process aims to seek consensus to a predetermined level via iterative rounds of scoring. Scorers, typically experts in a given field, can re-consider their scores based on the decision of others and also provide details/evidence to help justify their chosen score/inform others.

In our Delphi process there was evidence that in some instances scorers changed their scores based on the group response. Comments indicate that changes were typically due to recognition of additional information/misinterpretation rather than forcing consensus. It was, however, not always possible to ascertain the primary reason behind any change in scores (e.g. in light of additional information, previous misinterpretation or desire to conform to the group response). While there is the risk that such reiterative scoring may increase the chance of group thinking rather than self-thinking (Rowe, Wright & McColl, 2005), allowing participants the scope to re-think their scores based on feedback from others is thought to result in more accurate and credible responses (Rowe, Wright & McColl, 2005). Furthermore, as the Delphi technique is anonymous the desire to conform to the group response is thought to be less of an issue when compared to other group based elicitation processes (Martin et al., 2012; Mukherjee et al., 2015).

In our process we fed back to experts for each of our three geographical regions independently. Thus experts in Eastern Europe were provided with summary scores and comments from other experts in Eastern Europe. Through seeking consensus at the regional level, this process could reduce inter-country variation (this limitation is highlighted in the results section). The authors recognise that personal opinions/experience and inter-country differences exist, and we felt that it was important to capture this variation. Thus to reduce the risk of enforcing consensus and losing this source of variation, scoring was discontinued after round two. This deviates slightly from most Delphi exercises where rescoring typically occurs till consensus has been achieved.

### References

Martin, T.G., Burgman, M.A., Fidler, F., Kuhnert, P.M., Low-Choy, S., McBride, M. & Mengersen, K. (2012). Eliciting expert knowledge in conservation science. Conservation Biology, 26, 29–38.

Mukherjee, N., Huge, J., Sutherland, W.J., McNeill, J., Van Opstal, M., Dahdouh-Guebas, F. & Koedam, N. (2015). The Delphi technique in ecology and biological conservation: applications and guidelines. Methods in Ecology and Evolution, 6, 1097-1109.

Rowe, G., Wright, G. & McColl, A. (2005). Judgment change during Delphi-like procedures: the role of majority influence, expertise, and confidence. Technological Forecasting and Social Change, 72, 377–399.
