# Supplementary material for: A critical analysis of the potential for EU Common Agricultural Policy measures to support wild pollinators on farmland
Source: J Appl Ecol. 2020 Feb 16;57(4):681–94. doi: 10.1111/1365-2664.13572 (PMC7188321; doi:10.1111/1365-2664.13572)
Supplement: Supplementary file 8 [file JPE-57-681-s008.pdf]

Appendix S2: Additional References

Aguiar Jr, T.R., Rasera, K., Parron, L.M., Brito, A.G. & Ferreira, M.T. (2015). Nutrient removal effectiveness by riparian buffer zones in rural temperate watersheds: The impact of no-till crops practices. Agricultural Water Management, 149, 74-80.

Alford, D.V. (1969). A Study of the Hibernation of Bumblebees (Hymenoptera: Bombidae) in Southern England. The Journal of Animal Ecology, 38, 149-170.

Barczi, A., Tóth, T.M., Csanádi, A., Sümegi, P. & Czinkota, I. (2006). Reconstruction of the paleo-environment and soil evolution of the Csípo-halom kurgan, Hungary. Quaternary International, 156, 49-59.

Balzan, M. V., Bocci, G. & Moonen, A.C. (2014). Augmenting flower trait diversity in wildflower strips to optimise the conservation of arthropod functional groups for multiple agroecosystem services. Journal of Insect Conservation, 18(4), 713-728.

Berg, Å., Ahrné, K., Öckinger, E., Svensson, R. & Wissman, J. (2013). Butterflies in semi-natural pastures and power-line corridors–effects of flower richness, management, and structural vegetation characteristics. Insect conservation and diversity, 6(6), 639-657.

Bond, D.A. & Kirby, E.J.M. (1999). *Anthophora plumipes* (Hymenoptera: Anthophoridae) as a pollinator of broad bean (*Vicia faba major*). Journal of Apicultural Research, 38(3-4), 199-203.

Bond, D.A. & Kirby, E.J.M. (2001). Further observations of *Anthophora plumipes* visiting autumn-sown broad bean (*Vicia faba major*) in the United Kingdom. Journal of apicultural research, 40(3-4), 113-114.

Brandle, J.R., Hodges, L. & Zhou, X.H. (2004). Windbreaks in North American agricultural systems. In *New Vistas in Agroforestry* (pp. 65-78). Springer, Dordrecht.

Campbell, J.W., Hanula, J.L. & Waldrop, T.A. (2007). Effects of prescribed fire and fire surrogates on floral visiting insects of the blue ridge province in North Carolina. Biological Conservation, 134(3), 393-404.

Carreck, N.L. & Williams, I.H. (2002). Food for insect pollinators on farmland: insect visits to flowers of annual seed mixtures. Journal of Insect Conservation, 6, 13–23.

Carvell, C., Meek, W.R., Pywell, R.F. & Nowakowski, M. (2004). The response of foraging bumblebees to successional change in newly created arable field margins. Biological Conservation, 118, 327–339.

Carvell, C., Roy, D.B., Smart, S.M., Pywell, R.F., Preston, C.D. & Goulson, D. (2006). Declines in forage availability for bumblebees at a national scale. Biological Conservation, 132, 481–489.

Carvell, C., Meek, W.R., Pywell, R.F., Goulson, D. & Nowakowski, M. (2007). Comparing the efficacy of agri-environment schemes to enhance bumble bee abundance and diversity on arable field margins. Journal of Applied Ecology, 44, 29–40.

Cole, L.J., Brocklehurst, S., Harrison, W., Robertson, D. & McCracken, D.I. (2012). Riparian field margins: their potential to enhance biodiversity in intensively managed grasslands. Insect Conservation and Diversity, 5, 86-94.

Cole, L.J., Brocklehurst, S., Robertson, D., Harrison, W. & McCracken, D.I. (2015). Riparian buffer strips: Their role in the conservation of insect pollinators in intensive grassland systems. Agriculture, Ecosystem & Environment, 211, 207-220.

Cole, L.J., Robertson, D., Harrison, W., Baddeley, J.A., Walker, R.L, & Watson, C.A. (2018). The potential for Nitrogen-fixing crops to deliver foraging resources for insect pollinators. Aspects of Applied Biology, 138, 107-112.

Collett, T.S. & Graham, P. (2015). Insect navigation: do honeybees learn to follow highways?. Current Biology, 25(6), R240-R242.

Cowgill, S.E., Sotherton, N.W. & Wratten, S.D. (1993). The selective use of floral resources by the hoverfly *Episyrphus balteatus* (Diptera: Syrphidae) on farmland. Annals of Applied Biology, 122(2), 223-231.

Cranmer, L., McCollin, D. & Ollerton, J. (2012). Landscape structure influences pollinator movements and directly affects plant reproductive success. Oikos, 121(4), 562-568.

Davies, Z.G. & Pullin, A.S. (2007). Are hedgerows effective corridors between fragments of woodland habitat? An evidence-based approach. Landscape Ecology, 22, 333-351.

Delaplane K.S. & Mayer, D.F. (2000). *Crop Pollination by Bees*. CABI Publishing, Wallingford, UK and New York, USA.

De Groot, M. & Bevk, D. (2012). Ecosystem services and phenology of hoverflies (Diptera: Syrphidae) in Slovenian forest stand. Les, 64 (5), 123-128.

Dicks, L.V., Baude, M., Roberts, S.P., Phillips, J., Green, M. & Carvell, C. (2015). How much flower-rich habitat is enough for wild pollinators? Answering a key policy question with incomplete knowledge. Ecological Entomology, 40, 22–35. doi:10.1111/een.12226

Diekötter, T., Walther-Hellwig, K., Conradi, M., Suter, M. & Frankl, R. (2006). Effects of landscape elements on the distribution of the rare bumblebee species *Bombus muscorum* in an agricultural landscape. Biodiversity and Conservation, 15, 57–68.

European Commission (2014). *Commission Delegated Regulation (EU) No 639/2014 of 11 March 2014 supplementing Regulation (EU) No 1307/2013 of the European Parliament and of the Council establishing rules for direct payments to farmers under support schemes within the framework of the common agricultural policy and amending Annex X to that Regulation.* OJ L 181, 20.6.2014, 1–47, ELI: http://data.europa.eu/eli/reg_del/2014/639/oj

European Commission (2016a). *Review of greening after one year.* European Commission, Brussels.

European Commission (2016b). *Review of greening after one year. Annex 2: Initial results of the implementation of green direct payments by farmers.* European Commission, Brussels.

European Commission (2016c). *Review of greening after one year. Annex 3: Impact on the level playing-field arising from Member States' implementation choices.* European Commission, Brussels.

Féon, V. L., Blanchard, H., Martinière, D., Bretaud, J.F., Guinard, E., Henry, M., Pineau, C., Vaissière, B.E. & François, D. (2016). The habitat value of power line rights-of-way for pollinators (bees and butterflies) in agricultural landscapes. In Guinard, E. editor. *IENE 2016 - Integrating Transport Infrastructure with Living Landscapes*, Lyon, France.

Fischer, J., Stott, J. & Law, B.S. (2010). The disproportionate value of scattered trees. Biological Conservation, 143, 1564-1567.

Fliszkiewicz, M., Kusnierczak, A. & Szymas, B. (2015). Reproduction of the red mason solitary bee *Osmia rufa* (syn. *Osmia bicornis*) (Hymenoptera: Megachilidae) in various habitats. European Journal of Entomology, 112, 100.

Franzén, M. & Nilsson, S.G. (2008). How can we preserve and restore species richness of pollinating insects on agricultural land? Ecography, 31, 698-708.

Free, J.B. (1993). *Insect Pollination of Crops.* 2nd Enlarged Edition, Academic Press, London.

Gelling, M., Macdonald, D.W. & Mathews, F. (2007). Are hedgerows the route to increased farmland small mammal density? Use of hedgerows in British pastoral habitats. Landscape Ecology, 22, 1019-1032.

González-Varo J.P., J.C. Biesmeijer, R. Bommarco, S.G. Potts, O. Schweiger, H.G. Smith, I. Steffan-Dewenter, H. Szentgyörgyi, M. Woyciechowski & M. Vilà. (2013). Combined effects of global change pressures on animal-mediated pollination. Trends in Ecology and Evolution, 28, 524-530. DOI.10.1016/j.tree.2013.05.008

Goulson, D., Hanley, M.E., Darvill, B., Ellis, J.S. & Knight, M.E. (2005). Causes of rarity in bumblebees. Biological conservation, 122(1), pp.1-8.

Grass, I., Albrecht, J., Jauker, F., Diekötter, T., Warzecha, D., Wolters, V. & Farwig, N. (2016). Much more than bees—Wildflower plantings support highly diverse flower-visitor communities from complex to structurally simple agricultural landscapes. Agriculture, Ecosystems & Environment, 225, 45-53.

Haaland, C., Naisbit, R. E. & Bersier, L.F. (2011). Sown wildflower strips for insect conservation: a review. Insect Conservation and Diversity, 4(1), 60-80.

Hannon, L.E. & Sisk, T.D. (2009). Hedgerows in an agri-natural landscape: potential habitat value for native bees. Biological Conservation, 142, 2140-2154.

Hanula, J.L., Ulyshen, M.D. & Horn, S. (2016). Conserving Pollinators in North American Forests: A Review. Natural Areas Journal, 36, 427-439.

Heneberg, P., Bogusch, P. & Řezáč, M. (2016). Off-road motorcycle circuits support long-term persistence of bees and wasps (Hymenoptera: Aculeata) of open landscape at newly formed refugia within otherwise afforested temperate landscape. Ecological Engineering, 93,187-198.

Hoehn, P., Steffan-Dewenter, I. & Tscharntke, T. (2011). Relative contribution of agroforestry, rainforest and openland to local and regional bee diversity. Biodiversity and Conservation, 19, 2189-2200.

Holland, J.M., Smith, B.M., Storkey, J., Lutman, P.J. & Aebischer, N.J. (2015). Managing habitats on English farmland for insect pollinator conservation. Biological Conservation, *182*, 215-222.

Hovestadt, T., Mitesser, O., Poethke, A. & Holzschuh, A. (2018). Explaining the variability in the response of annual eusocial insects to mass-flowering events. Journal of Animal Ecology, early view. doi: 10.1111/1365-2656.129

Hua, F., Wang, X., Zheng, X., Fisher, B., Wang, L., Zhu, J., Tang, Y., Yu, D.W. & Wilcove, D.S. (2016). Opportunities for biodiversity gains under the world's largest reforestation programme. Nature Communications, 7, 12717.

Jha, S. & Vandemeer, J.H. (2010). Impacts of coffee agroforestry management on tropical bee communities. Biological Conservation, 143(6), 1423-1431.

Kasina, M., Hagen, M., Kraemer, M., Nderitu, J., Martius, C. & Wittmann, D. (2009). Bee pollination enhances crop yield and fruit quality in Kakamega, western Kenya. East African Agricultural and Forestry Journal, 75(1).

Kehinde, T. & Samways, M.J. (2012). Endemic pollinator response to organic vs. conventional farming and landscape context in the Cape Floristic Region biodiversity hotspot. Agriculture, Ecosystems and Environment, 146(1), 162–167.

Kirk, W.D.J. & Howes, F.N. (2012). *Plants for bees. International Bee Research Association.* Cardiff, UK.

Kleijn, D. & van Langevelde, F. (2006). Interacting effects of landscape context and habitat quality on flower visiting insects in agricultural landscapes. Basic and Applied Ecology, 7, 201-214.

Kleijn, D., Berendse, F., Smit, R., Gilissen, N., Smit, J., Brak, B. & Groeneveld, R. (2004), The ecological effectiveness of agri-environment schemes in different agricultural landscapes in The Netherlands. Conservation Biology, 18, 775-786.

Klein, A.M., Steffan-Dewenter, I., Buchori, D. & Tscharntke T. (2002). Effects of land-use intensity in tropical agroforestry systems on coffee flower-visiting and trap-nesting bees and wasps. Conservation Biology, 16, 1003-1014.

Larrieu, L., Cabanettes, A. & Sarthou, J.P. (2015). Hoverfly (Diptera: Syrphidae) richness and abundance vary with forest stand heterogeneity: Preliminary evidence from a montane beech fir forest. European Journal of Entomology, 112, 755-769.

Lazarina, M., Sgardelis, S.P. Tscheulin, T., Devalez, J., Mizerakis, V., Kallimanis, A.S., Papakonstantinou, S., Kyriazis, T. & Petanidou, T. (2017). The effect of fire history in shaping diversity patterns of the flower-visiting insects in post-fire Mediterranean pine forests. Biodiversity and Conservation, 26(1), 115-131.

Lázaro, A., Tscheulin, T., Devalez, J., Nakas, G. & Petanidou T. (2016a). Effects of grazing intensity on flower cover, pollinator abundance and diversity, and pollination services. Ecological Entomology, 41, 400–412.

Lázaro, A., Tscheulin, T., Devalez, J., Nakas, G., Stefanaki, A., Hanlidou, E. & Petanidou, T. (2016b). Moderation is best: effects of grazing intensity on pollination networks in Mediterranean communities. Ecological Applications, 26, 796-807.

Lichtenberg, E.M., Kennedy, C.M., Kremen, C., Batary, P., Berendse, F., Bommarco, R., Bosque-Pérez, N.A., Carvalheiro, L.G., Snyder, W.E., Williams, N.M. & Winfree, R. (2017). A global synthesis of the effects of diversified farming systems on arthropod diversity within fields and across agricultural landscapes. Global Change Biology, 11, 4946-57.

Lourenço, L. (2006). Paisagens de socalcos e Riscos Naturais em Vales do Rio Alva. Coimbra, Faculdade de Letras da Universidade de Coimbra, Núcleo de Investigação Científica de Incêndios Florestais, Colectâneas Cindínicas, VI, 192

Lye, G., Park, K., Osborne, J., Holland, J. & Goulson, D. (2009). Assessing the value of Rural Stewardship schemes for providing foraging resources and nesting habitat for bumblebee queens (Hymenoptera: Apidae). Biological Conservation, 142(10), 2023-2032.

Magrach, A., Holzschuh, A., Bartomeus, I., Riedinger, V., Roberts, S.P., Rundlöf, M., Vujić, A., Wickens, J.B., Wickens, V.J., Bommarco, R. & González-Varo, J.P. (2018). Plant–pollinator networks in semi-natural grasslands are resistant to the loss of pollinators during blooming of mass-flowering crops. *Ecography*, *41*(1), pp.62-74.

Manning, A.D., Fischer, J. & Lindenmayer, D.B. (2006). Scattered trees are keystone structures - implications for conservation. Biological Conservation, 132, 311-321.

Martins, D.J. (2013). People, plants and pollinators: uniting conservation, food security, and sustainable agriculture in East Africa. Conservation Biology: Voices from the Tropics, 27, 232-238.

McCracken, D.I., Cole, L.J., Harrison, W. & Robertson, D. (2012). Improving the farmland biodiversity value of riparian buffer strips: conflicts and compromises. Journal of Environmental Quality 41, 355-363.

Meyer, B., Jauker, F. & Steffan-Dewenter, I. (2009). Contrasting resource-dependent responses of hoverfly richness and density to landscape structure. Basic and Applied Ecology, 10(2), 178-186.

Moisan-DeSerres, J., Chagnon, M. & Fournier, V. (2015). Influence of windbreaks and forest borders on abundance and species richness of native pollinators in lowbush blueberry fields in Quebec, Canada. Canadian Entomologist, 147, 432-442.

Montero-Castaño A., FJ Ortiz-Sánchez & M Vilà. (2016). Mass flowering crops in a patchy agricultural landscape can reduce bee abundance in adjacent shrublands. Agriculture, Ecosystems & Environment, 223, 22-30.

Murray, T.E., Fitzpatrick, Ú., Byrne, A., Fealy, R., Brown, M.J.F. & Paxton, R.J. (2012). Local-scale factors structure wild bee communities in protected areas. *Journal of Applied Ecology*, 49, 998–1008. doi: 10.1111/j.1365-2664.2012.02175.x

Musters, C.J.M., van Alebeek, F., Geers, R.H.E.M., Korevaar, H., Visser, A. & de Snoo, G.R. (2009). Development of biodiversity in field margins recently taken out of production and adjacent ditch banks in arable areas. Agriculture, Ecosystems and Environment, 129, 131–139.

Naiman, R.J. & Decamps, H. (1997). The ecology of interfaces: riparian zones. Annual review of Ecology and Systematics, 28(1), 621-658.

Nicholls, C.I. & Altieri, M.A. (2013). Plant biodiversity enhances bees and other insect pollinators in agroecosystems. A review. Agronomy for Sustainable Development, 33(2), 257–274.

Nilsson, C., Xiong, S., Johansson, M.E. & Vought, L.B.M. (1999). Effects of leaf-litter accumulation on riparian plant diversity across Europe. Ecology, 80(5), 1770-1775.

Olson, D. M. & Wäckers, F. L. (2007). Management of field margins to maximize multiple ecological services. Journal of Applied Ecology, 44(1), 13–21. doi:10.1111/j.1365-2664.2006.01241.x

Osborne, J.L., Martin, A.P., Shortall, C.R., Todd, A.D., Goulson, D., Knight, M.E., Hale, R.J. & Sanderson, R.A. (2008). Quantifying and comparing bumblebee nest densities in gardens and countryside habitats. Journal of Applied Ecology, 45(3), 784-792.

Pafilis, P., Valakos, S., Konstantinidis, T., Foufopoulos, J., Theodosiou, A., Anastasiou, J., Karameta, E. & Sagonas, K. (2014). *Drystone walls as a biodiversity supporters. Final Report to Ioannis S. Latsis Foundation*, Athens.

Pérez-Bañón, S., Radenković, S., Vujić, A., Ståhls, G., Rojo, S., Grković, A. & Petanidou, T. (2016). *Brachyopa minima* (Diptera: Syrphidae), a new species from the Greek island

of Lesvos with notes on the biodiversity and conservation of the genus Brachyopa Meigen, in the Northern Aegean Islands. Zootaxa, 4072, 217–234.

Persson A.S. & Smith, H. (2013). Seasonal persistence of bumblebee populations is affected by landscape context. Agriculture, Ecosystems & Environment, 165, 201-209.

Petanidou T. (2015). *Terraces of the Aegean – the example of the Dodecanese*. Parisianou Scientific Publications, Athens.

Petanidou, T. & Ellis, W.N. (1996). Interdependence of native bee faunas and floras in changing Mediterranean communities. In: *The Conservation of Bees* – Linnean Society Symposium series No 18 (A. Matheson, S.L. Buchmann, C. O'Toole, P. Westrich, I.H. Williams, eds.), 201–226. Academic Press, London.

Pfiffner, L. & Müller, A. (2016). Wild bees and pollination. pp: 1-8. Research Institute of Organic Agriculture. FiBL.

Pierre, J., Le Guen, J., Delègue, M.P., Mesquida, J., Marilleau, R. & Morin, G. (1996). Comparative study of nectar secretion and attractivity to bees of two lines of spring-type faba bean (*Vicia faba* L var equina Steudel). Apidologie, 27(2), 65-75.

Pierre, J., Suso, M.J., Moreno, M.T., Esnault, R. & Le Guen, J. (1999). Diversité et efficacité de l'entomofaune pollinisatrice (Hymenoptera: eApidae) de la féverole (*Vicia faba* L.) sur 2 sites, en France et en Espagne. Annales de la Société Entomologique de France, 35:312-318.

Pollard E. & Yates, T.J. (1993). *Monitoring Butterflies for Ecology and Conservation*. Chapman & Hall, London, UK.

Potts, S.G., Petanidou, T., Roberts, S., O'Toole, C., Hulbert, A., Willmer, P. (2006). Plant–pollinator biodiversity and pollination services in a complex Mediterranean landscape. Biological Conservation, 129, 519–529.

Potts, S.G., Woodcock, B.A., Roberts, S.P.M., Tscheulin, T., Pilgrim, E.S., Brown, V.K. & Tallowin, J.R. (2009). Enhancing pollinator biodiversity in intensive grasslands. Journal of Applied Ecology, 46(2), 369-379.

Power, E.F., Jackson, Z. & Stout, J.C. (2016). Organic farming and landscape factors affect abundance and richness of hoverflies (Diptera, Syrphidae) in grasslands. Insect Conservation and Diversity, 9(3), pp.244-253.

Putra, R.E. & Nakamura, K. (2009). Foraging ecology of a local wild bee community in an abandoned Satoyama system in Kanazawa, Central Japan. Entomological Research, 39, 99-106.

Pywell, R.F., Warman, E.A., Carvell, C., Sparks, T.H., Dicks, L.V., Bennett, D., Wright, A., Critchley, C.N.R. & Sherwood, A. (2005). Providing foraging resources for bumblebees in intensively farmed landscapes. Biological Conservation, 121, 479–494.

Pywell, R.F., Warman, E.A., Hulmes, L., Hulmes, S., Nuttall, P., Sparks, T.H., Critchley, C.N.R. & Sherwood, A. (2006). Effectiveness of new agri-environment schemes in providing foraging resources for bumblebees in intensively farmed landscapes. Biological Conservation, 129(2), 192-206.

Reemer, M., Renema, W., Van Steenis, W., Zeegers, T., Barendregt, A., Smit, J.T., Van Veen, M.P., Van Steenis, J. & Van der Leij, L.J.J.M. (2009). De Nederlandse Zweefvliegen (Diptera: Syrphidae). *Nederlandse Fauna 8.* Nationaal Natuurhistorisch Museum Naturalis, Leiden.

Robertson, P.A. & Sotherton, N.W. (1992). Arable energy coppice as a wildlife habitat. In: Richards, G.E. (ed.) *Wood Energy and the Environment.* Harwell Laboratories, Oxford.

Rotheray, G.E. (1993). A colour guide to hoverfly larvae in Britain and Europe. Dipterists Digest 9:1-155.

Rubene, D., Schroeder, M. & Ranius, T. (2015). Diversity patterns of wild bees and wasps in managed boreal forests: Effects of spatial structure, local habitat and surrounding landscape. Biological Conservation, 184, 201-208.

Rundlöf, M., Persson, A.S., Smith, H.G. & Bommarco, R. (2014). Late-season mass-flowering red clover increases bumble bee queen and male densities. Biological Conservation, 172, 138–145. doi:10.1016/j.biocon.2014.02.027

Russell, K. N., Ikerd, H. & Droege, S. (2005). The potential conservation value of unmowed powerline strips for native bees. Biological Conservation, 124, 133-148.

Sage, R.B. (2008). High invertebrate biodiversity in willow short rotation coppice can be protected when controlling chrysomelid pests by using a spatially targeted insecticide application. In: *Proceedings Crop Protection in Northern Britain 2008:* 33-38. The Association for Crop Protection in Northern Britain.

Saunders, M.E., Luck, G.W. & Mayfield, M.M. (2013). Almond orchards with living ground cover host more wild insect pollinators. Journal of Insect Conservation, 17(5), 1011–1025.

Sayer, C.D. Andrews, K., Shilland, E., Edmonds, N., Edmonds-Brown, R., Patmore, I.R., Emson, D. & Axmacher, J.A. (2012). The role of pond management for biodiversity conservation in an agricultural landscape. Aquatic Conservation: Marine and Freshwater Ecosystems, 22, 626-638.

Scheper, J., Bommarco, R., Holzschuh, A., Potts, S.G., Riedinger, V., Roberts, S.P., Rundlöf, M., Smith, H.G., Steffan-Dewenter, I., Wickens, J.B., Wickens, V.J. & Kleijn, D. (2015). Local and landscape-level floral resources explain effects of wildflower strips on wild bees across four European countries. Journal of Applied Ecology, 52(5), 1165-1175.

Sebek, P., Vodka, S., Bogusch, P., Pech, P., Tropek, R., Weiss, M., Zimova, K. & Cizek, L. (2016). Open-grown trees as key habitats for arthropods in temperate woodlands: The

diversity, composition, and conservation value of associated communities. Forest Ecology and Management, 380,172-181.

Sladen, F.W.L. (1912). *The Humble Bee: Its Life History and How to Domesticate It.* Macmillan and Co. Ltd, London.

Smith, H., Feber, R.E. & MacDonald, D.W. (2015). From weed reservoir to wildlife source – redefining arable field margins. In MacDonald, D.W. & Feber, R.E. (Eds.) *Wildlife conservation on Farmland, Volume 1 – Managing for Nature on Lowland Farms*. Oxford University Press, Oxford, UK.

Southwick, E.E. & Buchmann, S.L., 1995. Effects of horizon landmarks on homing success of honeybees. The American Naturalist, 146, 748-764.

Steffan-Dewenter, I. & Tscharntke, T., 2001. Succession of bee communities on fallows. Ecography, 24(1), 83-93.

Suso, MJ., Bebeli, P. & Palmer, R. (2015). Reproductive Biology of Grain Legumes. In: Antonio M. De Ron (ed.) *Grain Legumes, Series Handbook of Plant Breeding.* Springer Science+Business Media, New York. pp. 365-399.

Sydenham, M.A.K., Eldegard, K. & Totland, O. (2014). Spatio-temporal variation in species assemblages in field edges: seasonally distinct responses of solitary bees to local habitat characteristics and landscape conditions. Biodiversity and Conservation, 23, 2393-2414.

Tamang, B. Andreu, M.G. & Rockwood, D.L. (2010). Microclimate patterns on the leeside of single-row tree windbreaks during different weather conditions in Florida farms: implications for improved crop production. Agroforestry Systems, 79, 111-122.

Toivonen, M., Herzon, I. & Kuussaari, M. (2016). Community composition of butterflies and bumblebees in fallows: niche breadth and dispersal capacity modify responses to fallow type and landscape. Journal of Insect Conservation, 20, 23-34.

Tropek, R., Cerna, I., Straka, J., Kadlec, T., Pech, P., Tichanek, F. & Sebek, P. (2014). Restoration management of fly ash deposits crucially influence their conservation potential for terrestrial arthropods. Ecological Engineering, 73, 45-52.

Tzilivakis, J., Warner, D.J., Green, A. & Lewis, K.A. (2015). *Guidance and tool to support farmers in taking aware decisions on Ecological Focus Areas.* Final report for Project JRC/IPR/2014/H.4/0022/NC. Joint Research Centre (JRC), European Commission.

Uchida, K. & Ushimaru, A. (2014). Biodiversity declines due to abandonment and intensification of agricultural lands: Patterns and mechanisms. Ecological Monographs, 84, 637-658.

Uchida, K. & Ushimaru, A. (2015). Land abandonment and intensification diminish spatial and temporal β-diversity of grassland plants and herbivorous insects within paddy terraces. Journal of Applied Ecology, 52, 1033-1043.

Vujić, A., Speight, M., de Courcy, M., Rojo, S., Ståhls, G., Radenković, S., Likov, L., Miličić, M., Pérez-Bañón, C., Falk, S. & Petanidou, T. (2020). *Atlas of the hoverflies (Diptera: Syrphidae) of Greece.* Brill Publishers, Leiden. Forthcoming.

Wagner, D. L., Ascher, J.S. & Bricker, N.K. (2014). A Transmission Right-of-Way as Habitat for Wild Bees (Hymenoptera: Apoidea: Anthophila) in Connecticut. Annals of the Entomological Society of America, 107, 1110-1120.

Westrich, P. (1996). Habitat requirements of central European bees and the problems of partial habitats. In: *The Conservation of Bees* (eds A. Matheson, S.L. Buchmann, C. O'Toole, P. Westrich, and I.H. Williams). Academic Press, London, UK, 1-16.

Willmer, P. (2011). *Pollination and Floral Ecology*. Princeton University Press, Princeton.

Wood, T.J., Holland, J.M., Hughes, W.O. & Goulson, D. (2015). Targeted agri-environment schemes significantly improve the population size of common farmland bumblebee species. Molecular ecology, 24(8), 1668-1680.

Wood, T.J., Holland, J.M. & Goulson, D. (2017). Providing foraging resources for solitary bees on farmland: current schemes for pollinators benefit a limited suite of species. Journal of Applied Ecology, 54(1), 323-333.
